# Persistent Organic Pollutants Modify Gut Microbiota–Host Metabolic Homeostasis in Mice Through Aryl Hydrocarbon Receptor Activation

**DOI:** 10.1289/ehp.1409055

**Published:** 2015-03-13

**Authors:** Limin Zhang, Robert G. Nichols, Jared Correll, Iain A. Murray, Naoki Tanaka, Philip B. Smith, Troy D. Hubbard, Aswathy Sebastian, Istvan Albert, Emmanuel Hatzakis, Frank J. Gonzalez, Gary H. Perdew, Andrew D. Patterson

**Affiliations:** 1 Center for Molecular Toxicology and Carcinogenesis, Department of Veterinary and Biomedical Sciences, The Pennsylvania State University, University Park, Pennsylvania, USA; 2 Chinese Academy of Sciences (CAS) Key Laboratory of Magnetic Resonance in Biological Systems, State Key Laboratory of Magnetic Resonance and Atomic and Molecular Physics, Wuhan Centre for Magnetic Resonance, Wuhan Institute of Physics and Mathematics, CAS, Wuhan, China; 3 Laboratory of Metabolism, National Cancer Institute, National Institutes of Health, Bethesda, Maryland, USA; 4 Bioinformatics Consulting Center, and; 5 Department of Chemistry, The Pennsylvania State University, University Park, Pennsylvania, USA

## Abstract

**Background:**

Alteration of the gut microbiota through diet and environmental contaminants may disturb physiological homeostasis, leading to various diseases including obesity and type 2 diabetes. Because most exposure to environmentally persistent organic pollutants (POPs) occurs through the diet, the host gastrointestinal tract and commensal gut microbiota are likely to be exposed to POPs.

**Objectives:**

We examined the effect of 2,3,7,8-tetrachlorodibenzofuran (TCDF), a persistent environmental contaminant, on gut microbiota and host metabolism, and we examined correlations between gut microbiota composition and signaling pathways.

**Methods:**

Six-week-old male wild-type and *Ahr*^–/–^ mice on the C57BL/6J background were treated with 24 μg/kg TCDF in the diet for 5 days. We used 16S rRNA gene sequencing, ^1^H nuclear magnetic resonance (NMR) metabolomics, targeted ultra-performance liquid chromatography coupled with triplequadrupole mass spectrometry, and biochemical assays to determine the microbiota compositions and the physiological and metabolic effects of TCDF.

**Results:**

Dietary TCDF altered the gut microbiota by shifting the ratio of Firmicutes to Bacteroidetes. TCDF-treated mouse cecal contents were enriched with *Butyrivibrio* spp. but depleted in *Oscillobacter* spp. compared with vehicle-treated mice. These changes in the gut microbiota were associated with altered bile acid metabolism. Further, dietary TCDF inhibited the farnesoid X receptor (FXR) signaling pathway, triggered significant inflammation and host metabolic disorders as a result of activation of bacterial fermentation, and altered hepatic lipogenesis, gluconeogenesis, and glycogenolysis in an AHR-dependent manner.

**Conclusion:**

These findings provide new insights into the biochemical consequences of TCDF exposure involving the alteration of the gut microbiota, modulation of nuclear receptor signaling, and disruption of host metabolism.

**Citation:**

Zhang L, Nichols RG, Correll J, Murray IA, Tanaka N, Smith PB, Hubbard TD, Sebastian A, Albert I, Hatzakis E, Gonzalez FJ, Perdew GH, Patterson AD. 2015. Persistent organic pollutants modify gut microbiota–host metabolic homeostasis in mice through aryl hydrocarbon receptor activation. Environ Health Perspect 123:679–688; http://dx.doi.org/10.1289/ehp.1409055

## Introduction

Obesity risk factors include changes in diet and lifestyle and, in some rare instances, can be explained by genetic predisposition (Jääskeläinen et al. 2013); however, these factors alone seem unlikely to explain the growing worldwide obesity epidemic. There is increasing evidence that chronic exposure to environmental chemicals through the diet, especially persistent organic pollutants (POPs), may promote the development of obesity and type 2 diabetes in humans (Lee et al. 2014). Of particular interest is the role of the aryl hydrocarbon receptor (AHR), which is bound and activated by a variety of POPs including coplanar polychlorinated biphenyls (PCBs) and halogenated aromatic hydrocarbons, in mediating the toxic effects of these compounds at high doses (Alaluusua and Lukinmaa 2006; Ren et al. 2011) and promoting obesity at low doses (Arsenescu et al. 2008; Howell and Mangum 2011). More recent evidence suggests that gut microbiota, which can be modulated by the AHR (Jin et al. 2014), may play a pivotal role in POP-induced obesity (Myre and Imbeault 2014).

The AHR is a xenobiotic sensor that was recently shown to be a pivotal regulator of gut and immune homeostasis (Monteleone et al. 2012). AHR agonists, including both endogenous (e.g., several tryptophan catabolites), dietary (e.g., indole-3-carbinol metabolite indolo[3,2b] carbazole), and xenobiotic [e.g., 2,3,7,8-tetrachlorodibenzo-*p*-dioxin (TCDD)] ligands, can dramatically impact host metabolism and immunity (Angrish et al. 2013; Bjeldanes et al. 1991; Claus et al. 2008; Zelante et al. 2013). For example, microbiota-derived tryptophan metabolites, including indole-3-aldehyde, were shown to modulate the AHR-IL-22 axis, thus impacting mucosal immune homeostasis in the gut (Zelante et al. 2013). Furthermore, alteration of gut microbiota through dietary manipulation and the presence of environmental contaminants may disturb physiological homeostasis, leading to various diseases such as obesity (Greenblum et al. 2012), diabetes (Mathis and Benoist 2012), and inflammatory bowel disease (Nagalingam and Lynch 2012). Recent studies suggest that interactions between gut microbiota and environmental toxicants may contribute in part to the development of obesity and diabetes (Snedeker and Hay 2012). Therefore, bacteria in the gut may be affected by dietary exposure to TCDD and other TCDD-like compounds, thus altering not only bacterial populations but also their inherent metabolic activity (Maurice et al. 2013). In the present study, we used TCDF instead of TCDD, given its considerably shorter half-life in rodents (DeVito and Birnbaum 1995), and we used doses higher than those of polychlorinated dibenzo-*p*-dioxins and dibenzofurans typically found in the diet of adults [0.3–3.0 pg toxic equivalents (TEQs)/kg body weight] (Nakatani et al. 2011; Schecter et al. 1994). Further, TCDF was provided through the diet over 5 days to represent the major route of exposure in humans (Jones and Bennett 1989).

In the present study we used a combination of 16S rRNA gene sequencing, metabolite profiling [ultra-performance liquid chromatography coupled with triplequadrupole mass spectrometry (UPLC-TQMS)], and ^1^H nuclear magnetic resonance (NMR)-based metabolomics to investigate the alteration of gut microbiota and the host metabolome in mice treated with TCDF through the diet. In addition, we investigated possible correlations between gut microbiota composition and signaling pathways, such as AHR and farnesoid X receptor (FXR), in response to TCDF exposure with the goal of identifying a specific host–microbiota signaling axis. Our findings provide new evidence that exposure to persistent environmental contaminants mediated through activation of the AHR strongly impacts the gut microbiota and host metabolism.

## Materials and Methods

*Animals and diets*. Animal experimental procedures were performed according to the National Institutes of Health (NIH) guidelines and were reviewed and approved by the Pennsylvania State University Institutional Animal Care and Use Committee. Mice were treated humanely and with regard for the alleviation of suffering. A total of twelve 8-week-old male C57BL/6J mice (*Ahr*^+/+^) were purchased from the Jackson Laboratory and housed together in groups of two to four mice per cage with a 12-hr light/dark cycle and constant temperature (23 ± 1°C) and humidity (45–65%); mice were allowed free access to food and water. Bedding material consisted of Alpha Dri (Shepherd Specialty Papers). After acclimatization for 1 week to normal flora in our facility, mice were randomly divided into two groups of six and trained to eat transgenic dough pills (Bio-Serve) that contained no TCDF (Cambridge Isotope Laboratories Inc.) for 4 days. Dough pills containing TCDF (acetone solution as vehicle) or acetone alone were prepared using tablet molds, and one pill uniformly contained 0.6 μg TCDF, thus providing a final dose of 24 μg/kg. In the TCDF studies, mice were fed the dough pills containing TCDF continuously for 5 days in the morning (one pill per mouse per day). Urine and feces were collected every other day (i.e., days 2, 4, and 6) over the TCDF treatment period. Blood, liver, intestine, cecum, and cecal content samples were collected immediately following CO_2_ asphyxiation on day 7. All samples were stored at –80°C until analysis. Ten male *Ahr*^–/–^ mice (C57BL/6J congenic mice) were obtained from C. Bradfield (University of Wisconsin) and inbred to maintain the line. All mice were killed by CO_2_ asphyxiation at 1300 hours, after they were transported to the laboratory.

*Quantitative real-time polymerase chain reaction (qPCR)*. RNA was extracted from frozen liver and intestine (~ 50 mg) using TRIzol reagent (Invitrogen). cDNA was synthesized from 1 μg of total RNA using Superscript II reverse transcriptase (Invitrogen), and the products were diluted to 1:10 before use in subsequent reactions. Gene-specific primers were used in each reaction, and all results were normalized to ribosomal protein *L13* or β*-actin* mRNA (for primer sequences, see Supplemental Material Table S1). qPCR assays were carried out using SYBR Green qPCR Master Mix on an ABI Prism 7900HT Fast Real-Time PCR sequence detection system (Applied Biosystems). The reactions were analyzed according to the ΔΔCT method. qPCR conditions were 40 cycles of 95°C for 20 sec, 95°C for 0.01 sec, 60°C for 20 sec, 95°C for 15 sec, 60°C for 15 sec, and 95°C for 15 sec.

*16S rRNA gene sequencing of the microbiota*. The bacteria in the cecal contents were extracted using the PowerSoil DNA Isolation Kit (MO BIO Laboratories Inc.). The PCR products (~ 1,000 bp) were purified using Agencourt AMPure technology (Beckman Coulter) as described for the Short Fragment Removal Procedure (Roche Diagnostics 2013). After purification, the products were quantified by both Qubit (Lifetech) and qPCR, using the KAPA Biosystems Library Quantification Kit (Kapa-Biosystems). Products were pooled based on molar concentrations, subjected to electrophoresis on a 1% agarose gel, and extracted. After purification with a QIAquick PCR Purification kit (QIAGEN), the quality and quantity of the preparations were assessed using a DNA 7500LabChip on the Agilent 2100 Bioanalyzer (Agilent Technologies) and Qubit quantification. Sequencing was performed using a quarter Picotiter Plate on a 454 Life Sciences Genome Sequencer FLX (Roche Diagnostics). 16S rRNA gene sequencing analysis was performed using the mothur platform, as previously described (Schloss et al. 2009).

*Bile acid profiling by TQMS*. Targeted analysis of bile acids was performed using an Acquity UPLC system coupled to a Waters Xevo TQS MS (Waters) with a C18 BEH column (2.1 × 100 mm, 1.7 μm; Waters). External bile acid standards, including cholic acid (CA), lithocholic acid (LCA), chenodeoxycholic acid (CDCA), deoxycholic acid (DCA), ursodeoxycholic acid (UDCA), α-muricholic acid (αMCA), β-muricholic acid (βMCA), ω-muricholic acid (ωMCA), glycocholic acid (GCA), glycochenodeoxycholic acid (GCDCA), taurocholic acid (TCA), taurodeoxycholic acid (TDCA), tauro chenodeoxycholic acid (TCDCA), taurolithocholic acid (TLCA), tauro-α-muricholic acid (TαMCA), and tauro-β-muricholic acid (TβMCA), were purchased from Sigma-Aldrich. The deuterated internal standards [cholic acid-2,2,4,4-D4 (CA-d4), lithocholic acid-2,2,4,4-D4 (LCA-d4), ursodeoxycholic acid-2,2,4,4-D4 (UDCA-d4), chenodeoxycholic acid-2,2,4,4 (CDCA-d4), and deoxycholic acid-2,2,4,4-D4 (DCA-d4)] were also obtained from Sigma-Aldrich. Tissues and feces were weighed (25 mg) and internal standards (5 μL, 1 μg/mL) were added before extraction with 500 μL ethanol by homogenization, followed by shaking at 60°C for 30 min. After cooling, the samples were incubated at 100°C for 3 min and centrifuged at 1,600 × *g* for 10 min at 4°C, and supernatants were transferred to a new tube. The pellets were extracted twice with 500 μL ethanol. After centrifugation at 11,200 × *g* for 1 min at 4°C, the combined supernatants were dried down and later dissolved with 500 μL methanol. Following vortexing and centrifugation, 300 μL of the supernatant was transferred to an autosampler vial. Analytes were detected by selected ion monitoring (SIM) or multiple reaction monitoring (MRM) and normalized by internal standard. Analytes of CA, LCA, UDCA, CDCA, and DCA were quantified with their respective deuterated internal standards. Others were quantified with CA-d4. The results were calculated according to individual standard curves established as follows: area_analyte_/area_internal std_. Retention times of analytes and their SIM/MRM are presented in Supplemental Material, Table S2.

*Fecal IgA and serum LAL (*Limulus *amebocyte lysate) measurements*. We obtained IgA mouse enzyme-linked immunosorbent assay (ELISA) kits from Abcam, and Pierce LAL chromogenic endotoxin quantitation kits and lipocalin-2 (LCN2) ELISA kits from Thermo Scientific. Fecal IgA, LCN2, and serum LAL were measured according to manufacturer instructions.

*Scanning electron microscopy (SEM)*. SEM images were used for observation of gut microbiota morphology, particularly to visualize bacteria associated with the intestinal epithelium. The ileum was opened and fixed with 2% glutaraldehyde overnight at 4°C. Samples were washed with 0.1 M sodium cacodylate buffer, pH 7.4, three times and then postfixed with 1% osmium tetroxide in 0.1 M sodium cacodylate buffer. The samples were washed and dehydrated through a graded ethanol series, then critical-point dried, mounted, and coated with 10 nm gold palladium alloy. Images were acquired on a JSM 5400 SEM (JEOL).

*TCDF reporter assay*. TCDF treatment in *Ahr*^–/–^ mice was confirmed using the Hepa1.1 mouse hepatoma reporter cell line. After killing the *Ahr*^–/–^ mice, 50 mg of liver was extracted using the Folch method (Folch et al. 1957), lyophylized, and reconstituted in 20 μL of DMSO; 0.1 μL of the DMSO-reconstituted extracted was used for the reporter assay. The Hepa 1.1 mouse hepatoma cell line containing the stably integrated pGudluc 1.1 luciferase reporter construct was obtained from M. Denison (University of California, Davis). The Hepa 1.1 mouse hepatoma cell line was maintained in α-modified essential media (Sigma-Aldrich) supplemented with 8% fetal bovine serum (Hyclone Laboratories) and 100 IU/mL penicillin/100 μg/mL streptomycin (Sigma-Aldrich). Cells were cultured at 37°C in a humidified atmosphere composed of 95% air and 5% CO_2_. The reporter cells (Hepa 1.1) were seeded in 12-well plates and cultured to approximately 90% confluence. The AHR responsiveness of extracts was examined after treatment for 4 hr, followed by lysing with 400 μL lysis buffer.

*Histopathology and clinical biochemistry*. Formalin-fixed liver tissue was embedded in paraffin, sectioned (3–4 μm), stained with hematoxylin and eosin (H&E), and examined under a microscope by a qualified liver pathologist (N.T.). Serum alanine transaminase (ALT) and alkaline phosphatase (ALP) were measured using the VetScan VS2 and the Mammalian Liver Profile rotor (Abaxis) according to the manufacturer’s instructions.

*NMR-based metabolomics experiment*. Sample preparation. Liver and intestinal tissues (~ 50 mg) were extracted three times with 600 μL of precooled methanol–water mixture (2/1, vol/vol) using the PreCellys Tissue Homogenizer (Bertin Technologies). Fecal and cecal content samples (~ 50 mg) were subjected to three consecutive freeze–thaws and directly extracted by precooled phosphate buffer with homogenization using the Precellys Tissue Homogenizer. After extraction, 550 μL of each extract was centrifuged and then transferred to a 5-mm NMR tube (for more detailed methods, see Supplemental Material, “NMR-based metabolomics experiment: Sample preparation”).

^1^H NMR spectroscopy. ^1^H NMR spectra of all the biological samples were recorded at 298 K on a Bruker Avance III 600 MHz spectrometer (operating at 600.08 MHz for ^1^H) equipped with a Bruker inverse cryogenic probe (Bruker Biospin). Typical one-dimensional NMR spectra were acquired for each of the samples employing the first increment of NOESY (Nuclear Overhauser effect spectroscopy) pulse sequence (NOESYPR1D). To facilitate NMR signal assignments, a range of two-dimensional (2D) NMR spectra were acquired and processed for selected samples (for more detailed methods, see Supplemental Material, “NMR-based metabolomics experiment: ^1^H NMR Spectroscopy”).

NMR data processing and multivariate data analysis. ^1^H NMR spectra were corrected manually for phase and baseline distortions, and spectral region δ 0.5–9.5 was integrated into regions with equal width of 0.004 ppm (2.4 Hz) using AMIX software (V3.8; Bruker-Biospin). Multivariate data analyses, including principal component analysis and orthogonal projection to latent structures with discriminant analysis (OPLS-DA), were performed on the NMR data using SIMCA-P+ software (version 13.0; Umetrics). To facilitate interpretation of the results, we performed color-coded correlation coefficient plots with back-transformation of the loadings generated from the OPLS-DA using an in-house–developed script for MATLAB (Mathworks) (see Supplemental Material, “NMR-based metabolomics experiment: Spectral data processing and multivariate data analysis”).

Data analysis. All experimental values are presented as mean ± SD. Graphical illustrations and statistical analysis were performed using GraphPad Prism (version 6.0; GraphPad). *p*-Values < 0.05 were considered as significant.

## Results

**Figure 1:**
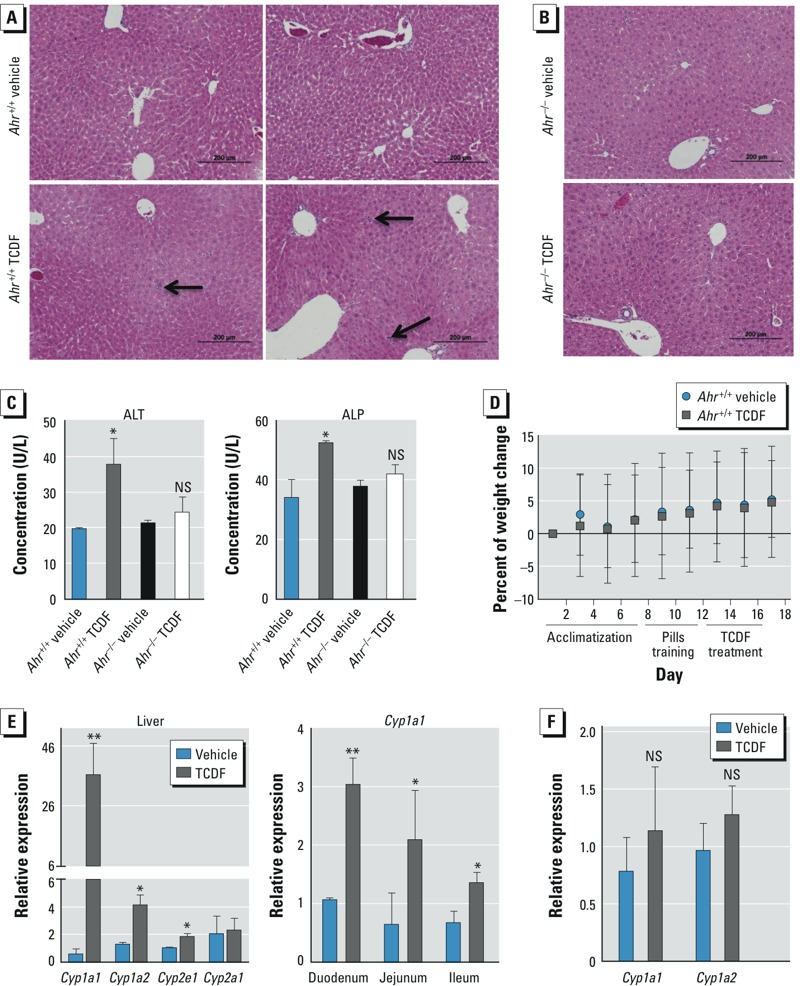
Xenobiotic responses in mice after dietary exposure to vehicle or TCDF (24 μg/kg). Light microscopic examination of H&E-stained liver sections from *Ahr*^+/+^ (*A*) and *Ahr*^–/–^ (*B*) mice; arrows indicate inflammatory foci (bars = 200 μm). (*C*) Serum concentrations of ALT (left) and ALP (right) from *Ahr*^+/+^ and *Ahr*^–/–^ mice. (*D*) Percent change in body weight of *Ahr*^+/+^ mice recorded every other day during the adaptation and treatment periods. (*E*) *Cyp1a1, Cyp1a2, Cyp2e1,* and *Cyp2a1* expression in the liver (left) and *Cyp1a1* mRNA expression in the intestine (right) of *Ahr*^+/+^ mice. (*F*) *Cyp1a1* and *Cyp1a2* mRNA expression in the liver of *Ahr*^–/–^ mice. Data are presented as mean ± SD; *n* = 5 or 6/group. NS, not significant. *p < 0.05, and **p < 0.01 by two-tailed Student’s *t*-test or Mann-Whitney test.

*Effects of dietary TCDF on liver enzymes and morphology*. The effect of sustained AHR activation on the gut was assessed using a potent AHR ligand, TCDF. *Ahr*^+/+^ and *Ahr*^–/–^ mice treated with dietary TCDF (24 μg/kg body weight) exhibited mild and no histopathological changes in the liver, respectively (Figure 1A,B). Mildly elevated serum ALT (19.3–37.5 U/L) and ALP (36.5–52.2 U/L) levels (Figure 1C), but no significant differences in body weight (Figure 1D), were observed in the *Ahr*^+/+^ mice after TCDF treatment, thus indicating minimal hepatic toxicity. Further, no evidence of steatosis, bile duct injury, or cellular degeneration was found, as is often observed with high-dose TCDD treatment (Ozeki et al. 2011). *Ahr*^–/–^ mice exhibited no significant changes in the liver enzymes ALT and ALP (Figure 1C). TCDF exposure in the *Ahr*^–/–^ mice was confirmed using dichloromethane extracts of liver and assessing the induction of AHR signaling in a Hepa 1.1 stable reporter cell line (see Supplemental Material, Figure S1). Transcriptional targets of AHR, including *Cyp1a1*, *Cyp1a2*, and *Cyp2e1,* were significantly induced in the liver and across the small intestine (Figure 1E) and were AHR dependent (Figure 1F). These observations suggest that the subsequent metabolic changes described below were specific to AHR activation and not due to overt liver toxicity.

**Figure 2:**
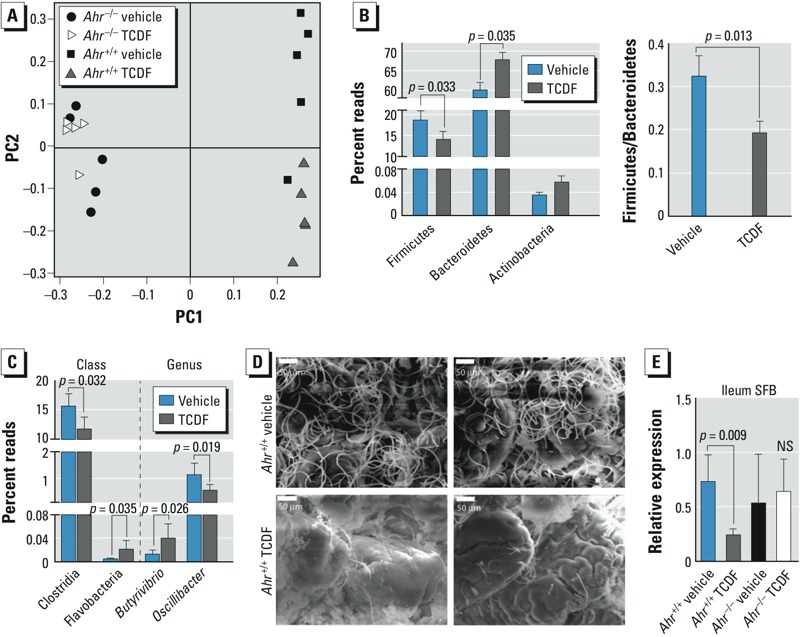
Effects of dietary TCDF on the morphology, population, and composition of gut microbiota of mice; animals were treated for 5 days with vehicle or TCDF (24 μg/kg) and sampled on day 7. (*A*) Weighted UniFrac principal coordinate analysis of the total population of the gut microbiome of cecal content from *Ahr*^+/+^ and *Ahr*^–/–^ mice. (*B*) 16S rRNA gene sequencing analysis of the cecal content of *Ahr*^+/+^ mice at the phylum level. (*C*) 16S rRNA gene sequencing analysis of cecal contents of *Ahr*^+/+^ mice at the class and genus levels. (*D*) Scanning electron microscopy images of ileum (bars = 50 μm) and (*E*) qPCR analysis of ileum SFB from *Ahr*^+/+^ and *Ahr*^–/–^ mice. Images in (*D*) represent replicates from two mice in each group. Data are presented as mean ± SD; *n* = 5 or 6/group. NS, not significant by two-tailed Student’s *t*-test or Mann-Whitney test.

*Effects of dietary TCDF on the morphology, population, and composition of gut microbiota*. Given recent evidence that the AHR is important for regulating gut homeostasis (Monteleone et al. 2013), and that in gut microbiota changed after oral dietary exposure to POPs (Snedeker and Hay 2012), we examined this relationship. Weighted UniFrac principal coordinate analysis (for assessing changes in abundance) of 16S rRNA sequencing results indicated that dietary TCDF induced a remarkable change in the overall population of gut microbiota (Figure 2A). Firmicutes and Bacteroidetes exhibited significant changes, with reduction of Firmicutes/Bacteroidetes ratio after dietary TCDF exposure (Figure 2B; see also Supplemental Material, Figure S2). Furthermore, cecal contents from TCDF-treated mice were enriched with *Butyrivibrio* spp. but depleted in *Oscillobacter* spp., compared with those from vehicle-treated mice (Figure 2C). Further, we observed an increase in the class Flavobacteria but a decrease in the class Clostridia. SEM images showed that dense segmented filamentous bacteria (SFB) formed a network of segmented filaments in the ileum of vehicle-treated mice, whereas they were dramatically depleted in the ileum of TCDF-treated mice (Figure 2D). qPCR analyses revealed that dietary TCDF significantly reduced SFB levels in the ileum, with no significant changes observed between TCDF-treated *Ahr*^–/–^ mice and vehicle-treated *Ahr*^–/–^ mice (Figure 2E).

**Figure 3:**
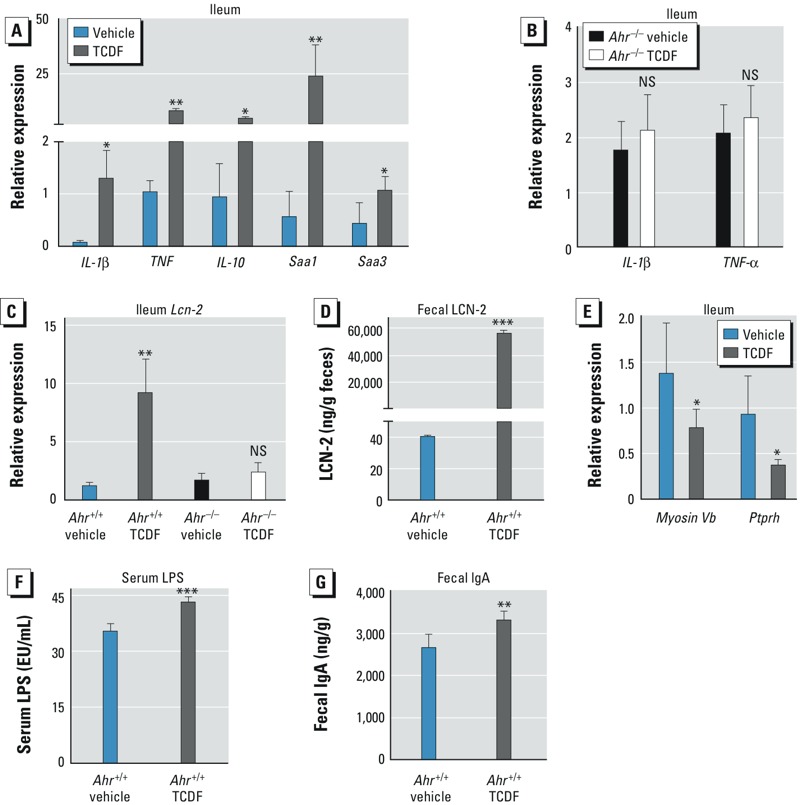
AHR-dependent inflammation in mice after dietary exposure to vehicle or TCDF (24 μg/kg). (*A*) qPCR analysis of inflammatory cytokine (*IL-1*β, *TNF*-α, *IL-10, Saa1,* and *Saa3*) mRNA expression in the ileum of *Ahr*^+/+^ mice. (*B*) *IL-1*β and *Tnf-*α expression in the ileum of *Ahr*^–/–^ mice. (*C*) qPCR analysis of *Lcn-2* mRNA expression in the ileum of *Ahr*^+/+^ and *Ahr*^–/–^ mice. (*D*) Quantification of fecal LCN2 in *Ahr*^+/+^ mice by ELISA. (*E*) qPCR analysis of *Myosin Vb* and *Ptprh* mRNA in the ileum of *Ahr*^+/+^ mice after TCDF treatment. (*F*) Quantification of serum LPS in *Ahr*^+/+^ mice. (*G*) Quantification of IgA in *Ahr*^+/+^ mice by ELISA. Data are presented as mean ± SD; *n* = 5/group. NS, not significant. *p < 0.05, **p < 0.01, and ***p < 0.001, by two-tailed Student’s *t*-test.

*Inflammatory signaling and immune responses*. TCDF led to a significant increase in mRNAs that encode factors in the ileum involved in inflammatory signaling, such as *Il-1*β, *Il-10*, *Tnf-*α, *Saa1*, and *Saa3* (Figure 3A); these changes were AHR dependent (Figure 3B). *Lcn-2* mRNA, encoding lipocalin-2, a sensitive biomarker for intestinal inflammation (Chassaing et al. 2012), was dramatically increased in the ileum (Figure 3C) and in the feces (Figure 3D) of mice exposed to TCDF. No significant change in *Lcn-2* mRNA (Figure 3C) was observed in the ileum of *Ahr*^–/–^ mice after TCDF treatment. Dietary TCDF resulted in a significant elevation of serum lipopolysaccharide (LPS) (Figure 3F) and fecal IgA (Figure 3G), and also resulted in decreased *Myosin Vb* and *Ptprh* in the ileum (Figure 3E), both of which are closely associated with gut permeability and the host immune system. Taken together, these results suggest that dietary TCDF triggers robust intestinal inflammation and inflammatory signaling in mice in an AHR-dependent manner.

**Figure 4:**
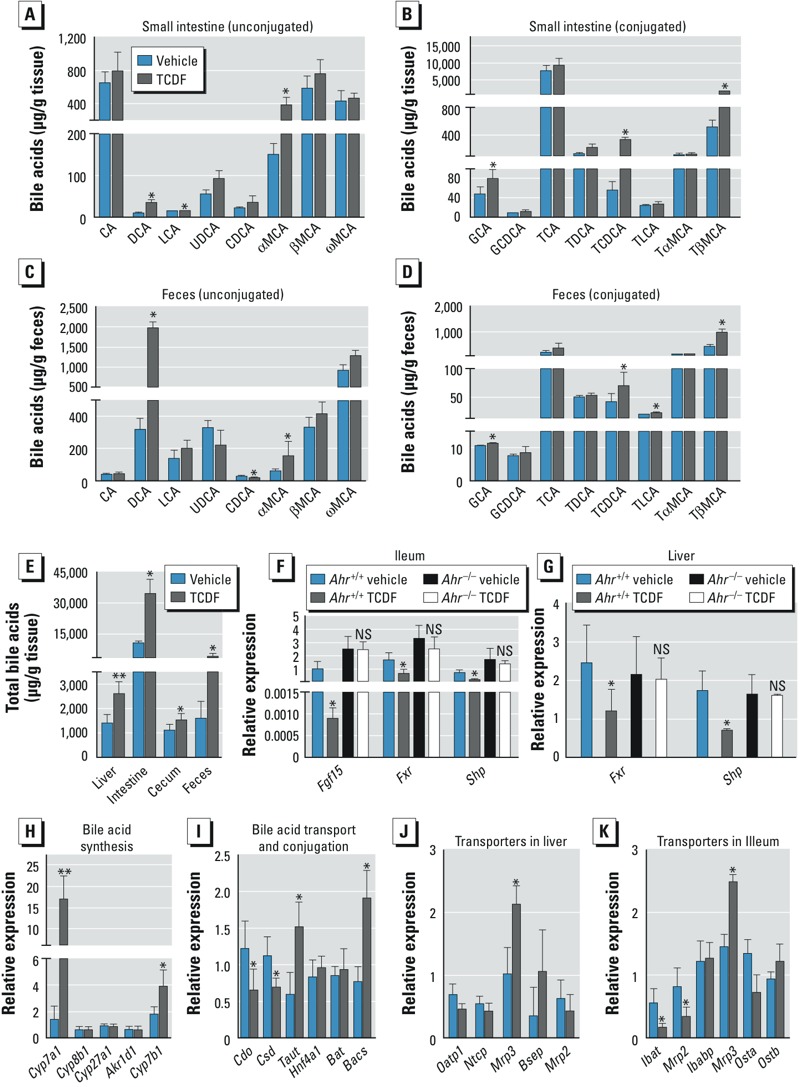
Bile acid metabolism in animals treated with vehicle or TCDF (24 μg/kg). Abbreviations: CA, cholic acid; CDCA, chenodeoxycholic acid; DCA, deoxycholic acid; G, glycine-conjugated; LCA, lithocholic acid; MCA, muricholic acid; NS, not significant; T, taurine-conjugated species; UDCA, ursodeoxycholic acid. Quantification of specific bile acids levels in intestinal tissue (*A,B*) and feces (*C,D*) throughout the enterohepatic circulation of *Ahr*^+/+^ mice. (*E*) Quantification of total bile acids in liver, intestine, cecum, and feces of *Ahr*^+/+^ mice; the bile acid profile in the small intestine shows the data from jejunum segment. qPCR analysis of *Fgf15*, *Fxr,* and *Shp* mRNAs in the ileum (*F*) and *Fxr* and *Shp* mRNA expression in the liver (*G*) of *Ahr*^+/+^ and *Ahr*^–/–^ mice. (*H*) qPCR analysis of *Cyp7a1*, *Cyp8b1*, *Cyp27a1*, *Akr1d1,* and *Cyp7b1* mRNAs in the liver of *Ahr*^+/+^ mice. (*I*–*K*) mRNA encoding bile acid transporters involved in taurine biosynthesis and bile acid conjugation in the ileum (*I*), and mRNA encoding bile acid transporters in the distal liver (*J*) and ileum (*K*) of vehicle- and TCDF-treated *Ahr*^+/+^ mice. Data are presented as mean ± SD; *n* = 6/group. See also Supplemental Material, Tables S1 and S2. *p < 0.05, and **p < 0.01, by two-tailed Student’s *t*-test.

*Bile acid metabolism*. It is well known that gut microbiota has profound effects on bile acid metabolism (Nicholson et al. 2012). To determine whether the composition of bile acids was affected by dietary TCDF exposure, we used UPLC-TQMS to examine bile acids and their levels in the liver, intestine, cecum, and feces, according to published methods (Jiang et al. 2015). Compared with vehicle-treated mice, TCDF-treated *Ahr*^+/+^ mice had significantly higher levels of DCA in the small intestine and feces (Figure 4A and 4C), and they also had significantly higher levels of three conjugated bile acids (GCA, TCDCA, and TβMCA) (Figure 4B,D) that were affected by gut microbial metabolism, as previously reported (Sayin et al. 2013). The size of the bile acid pool was significantly increased in TCDF-treated mice (Figure 4E). In addition, levels of LCA (intestine and cecum), CDCA (liver), and TLCA (liver and feces) in TCDF-treated mice were higher than those in vehicle-treated mice (see Supplemental Material, Figure S3A–E).

*Effects of dietary TCDF on gene expression profiles and FXR signaling*. FXR serves as a critical modulator of enterohepatic circulation and thus plays a key role in the regulation of bile acid synthesis and homeostasis (Claudel et al. 2005). TCDF treatment was associated with significant down-regulation of *Fxr* mRNA and its target gene small heterodimer partner (*Shp*) mRNA in both the ileum and liver of mice (Figure 4F,G). Intestinal fibroblast growth factor 15 (*Fgf15*) mRNA, which encodes a growth factor that is released by small intestine epithelial cells, was also markedly decreased by TCDF treatment (Figure 4F). In addition, no significant differences in the expression of *Fxr*, *Fgf15*, and *Shp* in the ileum (Figure 4F) or liver (Figure 4G) were observed between TCDF-treated and vehicle-treated *Ahr*^–/–^ mice. Taken together, these observations indicate that TCDF affects FXR signaling pathways through an AHR-dependent mechanism.

*Effects of dietary TCDF on gene expression profiles: genes associated with bile acid synthesis, conjugation, and reabsorption*. Next, we addressed the question of whether altered bile acid profiles in TCDF-treated *Ahr*^+/+^ mice were associated with changes in gene expression of enzymes involved in bile acid synthesis and/or transport. TCDF treatment resulted in a 10-fold elevation in the expression of *Cyp7a1* mRNA in the liver (Figure 4H), and CYP7A1 protein levels were also increased (see Supplemental Material, Figure S4). Expression of *Cyp7b1* mRNA was also increased by 1.8-fold in the liver (Figure 4H). In contrast, no significant change in *Cyp7a1* mRNA expression was observed in TCDF-treated *Ahr*^–/–^ mice (see Supplemental Material, Figure S3E). The expression of mRNAs encoded by other genes, such as *Cyp8b1*, *Cyp27a1*, and *Akr1d1*, all involved in bile acid synthesis, were not significantly affected by TCDF exposure (Figure 4H).

We observed a significant decrease in mRNAs encoding the taurine biosynthetic enzymes cysteine dioxygenase (CDO) and cysteine sulfinate decarboxylase (CSD), the rate-limiting enzyme for the conversion of cysteine to taurine (Bitoun and Tappaz 2000a, 2000b), in the livers of TCDF-treated *Ahr^+/+^* mice (Figure 4I). These observations were confirmed by the TCDF-induced down-regulation of taurine levels in gut tissues revealed by ^1^H NMR metabolomics (see Supplemental Material, Figure S6A–C and Table S3). However, expression of taurine transporter (*Taut*) mRNA, the main regulator of intracellular taurine levels (Bitoun and Tappaz 2000b), was up-regulated in the livers of TCDF-treated mice (Figure 4I), which may indicate an attempt to compensate for the reduced hepatic taurine content. We also noted a significant elevation in the expression of bile acid–coenzyme A synthetase (*Bacs*) mRNA—encoding an important enzyme involved in the process of bile acid conjugation with taurine—in the livers of TCDF-treated mice (Figure 4I). These results are in agreement with the observation of elevated taurine-conjugated bile acids.

For enterohepatic circulation of bile acids, both the hepatocyte and the enterocyte must efficiently transport bile acids. Thus, transporters play key roles in the transport and reabsorption of bile acids. In this study, the expression of genes involved in bile acid transport and homeostasis in liver and ileum were determined (Figure 4J–K). The multidrug-resistance protein (*Mrp3*) gene was significantly up-regulated in the livers of TCDF-treated *Ahr^+/+^* mice (Figure 4J). Previous studies reported that TCDD exposure resulted in up-regulation of *Mrp3* in mouse liver (Maher et al. 2005) and induced expression of inflammatory mediators as well as necrosis- and/or apoptosis-related genes (Hayashi et al. 2005). Expression of *Mrp2* and *Ibat* mRNAs (encoding apical bile acid transporters) were down-regulated, whereas *Mrp3* mRNA (encoding one of the basolateral bile acid transports) was up-regulated in the ileum of TCDF-treated mice (Figure 4K).

**Figure 5:**
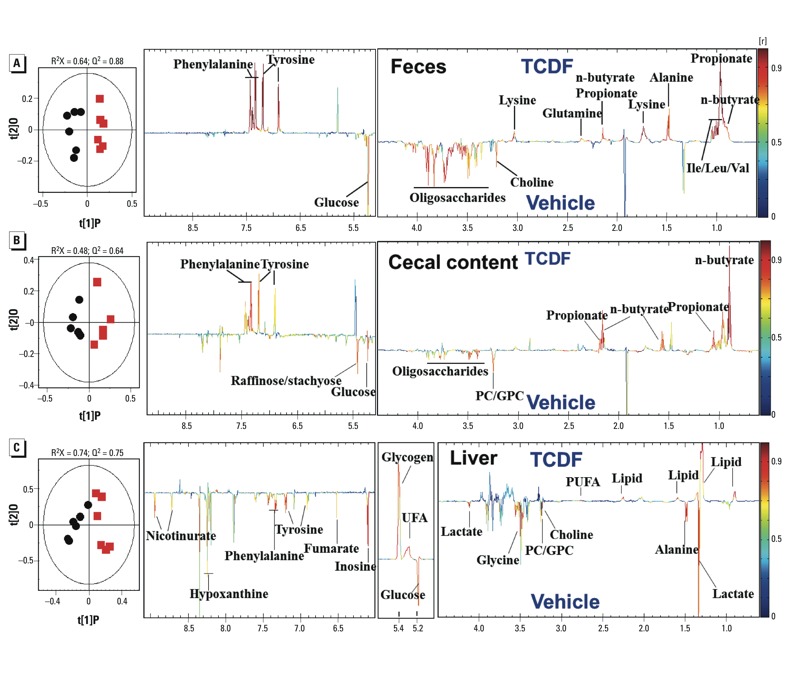
Host metabolism and the bacterial fermentation process in *Ahr*^+/+^ mice after dietary exposure to vehicle or TCDF (24 μg/kg). OPLS-DA scores (left) and coefficient-coded loadings plots for the models (right) from NMR spectra of aqueous fecal (*A*), cecal content (*B*), and liver extracts (*C*), discriminating between the vehicle (black circles) and TCDF-treated mice (red squares). These models are cross-validated with CV-ANOVA: *p* = 1.64 × 10^–3^, *p* = 0.033, and *p* = 0.0018 for feces, cecal content, and liver, respectively. Metabolite assignment is shown in Supplemental Material, Figure S5 and Table S4, and correlation coefficient values for the significantly changed metabolites are shown in Supplemental Material, Table S3.

*Effects of dietary TCDF on the host metabolome*. ^1^H NMR–based metabolomics coupled with multivariate statistical analysis was employed to evaluate the metabolic changes induced by TCDF exposure. Typical ^1^H NMR spectra of liver, fecal, and cecal content extracts obtained from vehicle- and TCDF-treated *Ahr*^+/+^ mice are presented in Supplemental Material, Figure S5A–F. Metabolite assignments were carried out as described previously (Tian et al. 2012; Wu et al. 2010) and confirmed with a series of 2D NMR experiments (see Supplemental Material, Table S4). To obtain the metabolic variations associated with different biological sample from vehicle- and TCDF-treated mice, we performed pair-wise OPLS-DA of data obtained from feces (Figure 5A), cecal contents (Figure 5B), liver (Figure 5C), and gut tissues, including duodenum, jejunum, ileum, and cecum (see Supplemental Material, Figure S6A–D). The model quality indicators (R^2^X and Q^2^; see Supplemental Material, Table S3) clearly showed that the extracts obtained from the biological matrices were distinctive in terms of their metabolite profiles. However, the Q^2^ values (0.007, –0.12, and 0.29 for the models of feces, cecal contents, and liver samples, respectively) of the OPLS-DA models of data from *Ahr*^–/–^ mice revealed no significant differences between TCDF and vehicle treatment (see Supplemental Material, Figure S6E–G and Table S5). These observations were further supported by results from the model evaluation with analysis of variance of the cross-validated residuals (CV-ANOVA) (*p* < 0.05) and permutation test for the OPLS-DA models (see Supplemental Material, Figure S7 and Table S5). The metabolites with statistically significant contributions between TCDF- and vehicle-treated groups are identified in the corresponding color-coded coefficient plots (Figure 5A–C; see also Supplemental Material, Figure S6A–D), and the correlation coefficient values were also tabulated (see Supplemental Material, Table S3). Compared with the vehicle-treated mice, dietary TCDF significantly elevated the levels of the short chain fatty acids (SCFAs) propionate and *n*-butyrate, but significantly decreased the levels of oligosaccharides and glucose in feces and cecal contents (Figure 5A,B; see also Supplemental Material, Table S3). Reliable assignments of *n*-butyrate and propionate were further confirmed by 2D ^1^H-^1^H total correlation spectroscopy (TOCSY) NMR spectroscopy (see Supplemental Material, Figure S8). Similar changes in SCFAs and oligosaccharides were also obtained from calculating their relative concentration from the NMR peaks integration to internal standard (sodium 3-trimethylsilyl [2,2,3,3-d4] propionate; TSP-d4) in feces and cecal contents of the TCDF-treated wild-type mice against those from the respective controls (see Supplemental Material, Figure S9A,B). However, no significant differences in the levels of SCFAs and oligosaccharides were observed in feces and cecal content between TCDF-treated and vehicle-treated *Ahr*^–/–^ mice (see Supplemental Material, Figure S9A,B), supporting the dependence on AHR status. Significant increases in the expression of the G-protein–coupled receptors (*Gpr41* and *Gpr43*) were found in the colons of TCDF-treated mice (see Supplemental Material, Figure S10A).

Dietary TCDF significantly elevated the levels of lipid, unsaturated fatty acid, and glycogen in the livers and significantly decreased the levels of glucose, lactate, fumarate, a range of amino acids, nucleotide metabolites (e.g., inosine and hypoxanthine), nicotinurate, and cell membrane–related metabolites [choline, phosphatidylcholine (PC), and glycerylphosphorylcholine (GPC)] in comparison with the vehicle-treated mice (Figure 5C). However, no significant change in lipid or glucose metabolism was observed in the liver of *Ahr*^–/–^ mice after TCDF treatment (see Supplemental Material, Figure S6G). Down-regulation of *Gck*, *G6pase*, *Glut2,* and *Pepck* expression was found in the livers of TCDF-treated *Ahr*^+/+^ mice (see Supplemental Material, Figure S10B).

In gut tissues, dietary TCDF exposure also resulted in a significant elevation of amino acids, including branched chain amino acids (leucine, isoleucine, and valine), lysine, glutamine, tyrosine, and phenylalanine, but reduction of taurine, choline, PC, GPC, and some nucleic acids such as hypoxanthine and uridine (see Supplemental Material, Figure S6A–D and Table S3).

## Discussion

Historically, POPs have been studied for their ability to cause adverse effects, including liver toxicity (Lee et al. 2014). We anticipate, however, that because most exposure to POPs occurs through the diet, the host gastrointestinal tract and gut microbiota are likely to be exposed to and modulated by POPs. In the present study, TCDF treatment increased gut inflammation, modulated the gut microbiota population, increased bacterial fermentation, and had a profound impact on host metabolism in an AHR-dependent manner.

Based on allometric scaling calculations, the TCDF dose (24 μg/kg body weight) used in this study is equivalent to approximately 3,000 ng/kg body weight in humans. Several epidemiological studies have reported that TCDD blood levels were, on average, 1,434 ng/kg (range 301–3,683 ng/kg) among workers involved in the remediation of the 2,4,5-trichlorophenol reactor accident in Seveso, Italy (World Health Organization 1998). Accounting for the reduced toxicity of TCDF compared with TCDD (the toxic equivalency factor for TCDF is 0.1), the dose used in the present study is considerably higher than the estimated daily oral intake of polychlorinated dibenzo-*p*-dioxins and dibenzofurans for adults (0.3–3.0 pg TEQ/kg body weight) (Nakatani et al. 2011; Schecter et al. 1994). Furthermore, we have not accounted for total body burden, and as mentioned above, the absorbed TCDF dose is considerably higher than what is expected from normal dietary intakes. However, our study reinforces the idea that gut microbiota are important targets and/or mediators of the toxicologic response and helps to establish possible end points for studies using lower doses of TCDF.

One of the most prominent findings in our investigation was the significant alteration of gut microbiota by TCDF and changes in co-metabolites of the host and gut microbiota, including bile acids and SCFAs. Within the intestinal microbiota, at the phylum level, TCDF treatment significantly shifted the microbial community from Firmicutes toward Bacteroidetes, indicating that the total population of the gut microbiota was modulated by TCDF. Similar observations have been observed with oral exposure to PCBs in mice, which interestingly, could be reversed with voluntary exercise (Choi et al. 2013). The predominant members, Firmicutes and Bacteroidetes, provide key metabolic functions to their host (Hooper et al. 2002), as well as important developmental (Stappenbeck et al. 2002) and immunologic properties (Hooper et al. 2003; Mazmanian et al. 2005). Further, TCDF treatment enriched *Butyrivibrio* spp., common butyrate-producing gut microbes, that are able to ferment a wide range of sugars and cellodextrins (Russell 1985). The increased levels of *Butyrivibrio* spp. was confirmed by the activation of fermentation by TCDF treatment, which induced significant depletion of glucose and oligosaccharides coupled with elevation of SCFAs such as butyrate and propionate in feces and cecal contents. Interestingly, certain members of the class Flavobacteria, which includes *Flavobacterium* spp., were reported to possess dehalogenase activity, and although it has not been demonstrated in isolates derived from the mammalian gut, they may contribute to the metabolism of TCDF and other halogenated compounds (Xun et al. 1992). A clear role for *Oscillibacter* spp. has not been established, but has been reported to be associated with the development of fatty liver, an effect observed with TCDD exposure (Angrish et al. 2013; Matsubara et al. 2012). Members of the Clostridia class and *Lactobacillus* spp. were reported to be associated with strong enzymatic activity of bile salt hydrolase (Li et al. 2013), and the elevation of DCA, αMCA, and TβMCA suggests that TCDF remodeling of gut microbiota contributes to changes in bile acid pools in the gut and liver (Jin et al. 2014).

After TCDF treatment of mice, SFB were drastically depleted, thus providing further evidence that TCDF impacted the gut microbiota. One of the most outstanding features of SFB as commensal bacteria is their specific attachment to host epithelial cells in the terminal ileum (Caselli et al. 2010). Previous studies demonstrated that SFB were present only in mice that had Th17 cell–inducing microbiota, which contain relatively abundant CD4^+^, IFNγ^+^, and CD25^+^Foxp3^+^ cells (Ivanov et al. 2009). SFB lead to an increase in the relative proportions of Th17 cells and Th17 cell cytokines, which provide protection against mucosal infection through modulation of the immune response of the host. Furthermore, other high-affinity ligands of AHR, TCDD and 6-formylindolo [3,2-b] carbazole (FICZ), have been shown to correlate with an increased frequency of Th17 cells (Hautekiet et al. 2008; Marshall and Kerkvliet 2010). Thus, TCDF exposure may be associated with heightened immune response that is dependent on AHR status. Additional studies focused on the direct impact of TCDF and other POPs on the gut microbiota are indeed warranted and should shed some light on how these compounds might influence the gut microbiota population directly.

Supporting the overall assertion that dietary TCDF exposure leads to a broad inflammatory response in mice is the significant elevation of fecal IgA, fecal LCN-2, serum LPS, and mRNA expression of intestinal innate immune factors including *Il-1*β, *Il-10*, *Tnf-*α, *Saa1,* and *Saa3* reported here. One possible explanation for these results is that the AHR plays a direct role in the regulation of inflammatory gene expression. There is support for this notion from studies that found functional dioxin response elements in the *Il-6* promoter that are capable of synergistically increasing transcription in the presence of an inflammatory signal and an AHR ligand (DiNatale et al. 2010). The binding of the AHR to the *Il-6* promoter results in jettison of HDAC1 (histone deacetylase 1) from the promoter, leading to increased NF-κB (nuclear factor-κB) occupation and acetylation. Whether the same type of combinatorial regulation occurs on other inflammatory genes remains to be explored. IgA, a major class of immunoglobulin, is produced in mucosal tissues and was reported to reduce intestinal proinflammatory signaling and bacterial epitope expression when binding to the commensal *Bacteroides thetaiotaomicron* (Peterson et al. 2007). This suggests that IgA inhibits innate immune responses and regulates the composition and the function of gut microbiota (Macpherson et al. 2012). LPS is found in the outer membrane of gram-negative bacteria, contributes greatly to the structural integrity of the bacteria, acts as an endotoxin, and elicits strong immune responses in animals. LPS can mediate an *IL-10–*dependent inhibition of CD4 T-cell expansion and function by up-regulating levels of PD-1 (programmed cell death protein 1) on monocytes, which leads to IL-10 production by monocytes after binding of PD-1 by PD-L (Said et al. 2010). Previous studies have shown that LPS in the portal vein or translocation of bacteria promotes intestinal fibrogenesis via TLR4 (toll-like receptor 4), predominantly from gram-negative bacteria (Chan et al. 1997; Lin et al. 1995). In the present study, an elevated level of serum LPS would suggest an increase in gut permeability or altered immune surveillance, leading to higher levels of inflammatory signaling in TCDF-treated mice compared with vehicle-treated mice. In addition, levels of LCN-2, a sensitive and dynamic noninvasive biomarker for intestinal inflammation, are dramatically increased after TCDF exposure (Chassaing et al. 2012). It is important to note that the observed innate immune responses in mice induced by TCDF required AHR expression. Whether the heightened host inflammatory signaling observed through potent AHR activation leads to changes in the gut microbiota, which in turn causes additional alterations in gut homeostasis, remains to be investigated.

Another prominent finding in the present study was the profound changes of the host metabolome induced by TCDF. In particular, TCDF induced significant reduction of glucose and oligosaccharides with elevation of SCFAs such as *n*-butyrate and propionate in feces and cecal content extracts, indicating activation of bacterial fermentation by TCDF. SCFAs are clearly one of the most important gut microbial products and affect a range of host processes including energy utilization, host–microbe signaling, gut composition, and motility (Nicholson et al. 2012). Schwiertz et al. (2010) reported that obesity is linked to the composition of human microbiota and the production of SCFAs, providing an additional source of energy for the body. In addition to being energy sources, SCFAs control colonic gene expression by inhibiting the enzyme HDAC and metabolic regulation by signaling through G-protein–coupled receptors, such as GPR41 and GPR43 (Tremaroli and Bäckhed 2012). Significant elevation of colonic *Gpr41* and *Gpr43* mRNAs further indicated activation of bacterial fermentation by TCDF.

Significantly elevated levels of lipid and unsaturated fatty acid in the liver of TCDF-treated mice indicate hepatic lipogenesis, which appears to be a common cellular response to a number of liver-toxic compounds such as allyl formate (Yap et al. 2006), acetaminophen (Coen et al. 2003), and aflatoxin-B1 (Zhang et al. 2011). Further, in the present study, depletion of hepatic glucose and lactate coupled with elevation of glycogen in TCDF-treated mice (Figure 5) suggests that one of the consequences of TCDF exposure is inhibition of gluconeogenesis and glycogenolysis. Similar observations were reported in the case of suppression of hepatic gluconeogenesis by TCDD through the AHR target gene *TiPARP* [TCDD-inducible poly(ADP-ribose) polymerase; Diani-Moore et al. 2010]. We also consistently observed markedly decreased expression and activity of *Pepck*, *Gck*, *G6pase*, and *Glut2*, all enzymes that control gluconeogenic flux in the liver of TCDF-treated mice. Interestingly TCDF exposure influenced the FXR signaling pathway, but the connection with AHR remains unclear. Further studies using tissue-specific *Ahr*-knockout mice should yield important insights into how AHR status in different tissues (e.g., liver or intestinal epithelium) impacts the gut microbiota.

## Conclusions

Dietary TCDF induced alterations in the gut microbiota accompanied by modulation of bile acid metabolism and host immune responses. Depletion of Clostridia by TCDF was associated with accumulation of DCA, which is involved in liver cancer (Yoshimoto et al. 2013). The increases in TCDCA and TβMCA by TCDF were associated with the inhibition of the FXR signaling pathway. Furthermore, through activation of AHR signaling, dietary TCDF altered many host metabolic pathways involved in hepatic lipogenesis, gluconeogenesis and glycogenolysis, bacterial fermentation, and amino acid and nucleic acid metabolism. These findings provide new evidence that exposure to persistent environmental contaminants strongly impacts the gut microbiota–host metabolic axis in mice.

## Supplemental Material

(1.9 MB) PDF
