# Supplementary material for: Persistent Organic Pollutants Modify Gut Microbiota–Host Metabolic Homeostasis in Mice Through Aryl Hydrocarbon Receptor Activation
Source: Environ Health Perspect. 2015 Mar 13;123(7):679–88. doi: 10.1289/ehp.1409055 (PMC4492271; doi:10.1289/ehp.1409055)
Supplement: (1.9 MB) PDF [file ehp.1409055.s001.acco.pdf]

Environ Health Perspect
doi: 10.1289/ehp.1409055

Note to Readers: ***EHP*** **strives to ensure that all journal content is accessible to all readers. However, some figures and Supplemental Material published in** ***EHP*** **articles may not conform to 508 standards due to the complexity of the information being presented. If you need assistance accessing journal content, please contact ehp508@niehs.nih.gov. Our staff will work with you to assess and meet your accessibility needs within 3 working days.**

Supplemental Material

Persistent Organic Pollutants Modify Gut Microbiota–Host Metabolic Homeostasis in Mice Through Aryl Hydrocarbon Receptor Activation

Limin Zhang, Robert G. Nichols, Jared Correll, Iain A. Murray, Naoki Tanaka, Philip Smith, Troy D. Hubbard, Aswathy Sebastian, Istvan Albert, Emmanuel Hatzakis, Frank J. Gonzalez, Gary H. Perdew, and Andrew D. Patterson

Table of Contents

Materials and Methods

NMR-based metabolomics experiment

Sample preparation

^{1}H NMR Spectroscopy

Spectral data processing and multivariate data analysis

Figure S1. AHR-null liver extract reporter assay. AHR-responsiveness of extracts was examined using hepatoma reporter line, Hepa 1.1. Reporter cells were treated with 0.1 µL of control or TCDF liver extracts for 4 h. Data represent mean±S.E.M (n=5), T-test parameters: Unpaired, Two tailed, p-value < 0.001 (***).

Figure S2. (A) qPCR analysis of mRNA levels of bacterial *Firmicutes* and *Bacteroidetes* in the cecal content of vehicle and TCDF-treated *Ahr*$^{+/+}$ mice. (B-D) 16S rRNA gene sequencing

analysis at the phylum and genus level of the cecal content. Data are presented as mean ± s. d, n = 6 and 5 per group for *Ahr*$^{+/+}$ and *Ahr*$^{-/-}$ mice, respectively; *p < 0.05, **p < 0.01, NS means no significance, two-tailed Student's t-test.

Figure S3. (A-D) Quantification of specific bile acids levels in liver and cecum of vehicle and TCDF-treated *Ahr*$^{+/+}$ mice (24 μg kg-1) by UPLC-TQMS. (E) qPCR analysis of mRNA levels of *Cyp7a1*, *Fxr* and *Shp* in the liver of vehicle and TCDF-treated *Ahr*$^{-/-}$ mice. Data are presented as mean ± s. d, n = 6 and 5 per group for *Ahr*$^{+/+}$ and *Ahr*$^{-/-}$ mice, respectively; *p < 0.05, **p < 0.01, NS, no significance, two-tailed Student's t-test. See also Table S1 and 2.

Figure S4. Western blot of Cyp7a1 and Actin levels in the liver.

Figure S5. Representative 600 MHz ^{1}H NMR spectra of liver (A and B), fecal (C and D) and cecal content (E and F) aqueous extracts from vehicle (B, D and F) and TCDF treated group (A, C and E). The regions of δ 6.1-9.20 and δ 0.6-3.1 in the liver spectra was vertically expanded 16 times and 4 times compared with the region of δ 3.1-4.7, respectively. The regions of δ 6.1-9.4 in the fecal aqueous extracts spectra were vertically expanded 16 times compared with the region of δ 0.5-4.5. The regions of δ 6.1-9.0 in the cecal content aqueous extracts spectra were vertically expanded 16 times compared with the region of δ 0.6-4.4. Keys: 1, lipid; 2, isoleucine; 3, leucine; 4, valine; 5, D-3-hydroxybutyrate; 6, lactate; 7, alanine; 8, acetate; 9, n-butyrate; 10, propionate; 11, threonine; 12, glutamate; 13, glutamine; 14, glutathione; 15, arginine; 16, proline; 17, creatine; 18, choline; 19, phosphorylcholine; 20, glycerophosphocholine; 21, β-glucose; 22, α-glucose; 23, unsaturated fatty acid; 24, TMAO; 25, tyrosine; 26, histidine; 27, phenylalanine; 28, formate; 29, betaine; 30, glycogen; 31, bile acid; 32, lysine; 33, N-acetyl aspartate; 34, oligosaccharides; 35, succinate; 36, taurine; 37, glycine; 38, inosine; 39, uridine; 40, fumarate; 41, nicotinurate; 42, adenosine; 43, uracil; 44, α-galactose; 45, α-arabinose; 46, α-xylose; 47, hypoxanthine; 48, glucose & amino acids; 49, ethanol; 50, pyruvate; 51,TMA; 52, raffinose; 53, stachyose; 54, methanol; 56, urocanate; 57, adenine; 58, α-ketoglutarate. See also Table S4.

Figure S6. O-PLS-DA scores (left) and coefficient-coded loadings plots for the models (right) from NMR spectra of aqueous duodenum (A), jejunum (B), ileum (C), and cecum (D) extracts from the vehicle and TCDF-treated *Ahr*$^{+/+}$ mice and fecal (E), cecal content (F) and liver (G) extracts from vehicle and TCDF-treated *Ahr*$^{-/-}$ mice.

Figure S7. Cross-validation with permutations test plots (200 permutations) for the PLS-DA models constructed from ^{1}H NMR data of liver (A, *Ahr*$^{+/+}$; B, *Ahr*$^{-/-}$), cecal content (C, *Ahr*$^{+/+}$; D, *Ahr*$^{-/-}$), fecal (E, *Ahr*$^{+/+}$; F, *Ahr*$^{-/-}$), duodenum (G), jejunum (H), ileum (I), and cecum (J) aqueous extracts from vehicle and TCDF-treated mice.

Figure S8. Two dimensional (2D) ^{1}H-^{1}H total correlation spectroscopy (TOCSY) for the identification of n-butyrate and propionate related to Figure 5A and B. The cross peaks of n-butyrate and propionate are highlighted with dotted and solid lines, respectively.

Figure S9. Measurements of n-butyrate and propionate concentration from NMR peaks integration relative to internal standard TSP in the cecal content (A) and fecal extracts (B) obtained from $Ahr^{+/+}$ and $Ahr^{-/-}$ vehicle and TCDF-treated mice. Data are presented as mean ± s. d, n = 6 and 5 per group for $Ahr^{+/+}$ and $Ahr^{-/-}$ mice, respectively; ; $*p < 0.05$, $**p < 0.01$, NS, no significance, two-tailed Student's t-test.

Figure S10. qPCR analysis of mRNA levels of *Gpr41* and *Gpr43* expression in the colon (A) and *Gck*, *G6pase*, *Glut2* and *Pepck* expression in the liver of $Ahr^{+/+}$ vehicle and TCDF-treated $Ahr^{+/+}$ mice. Data are presented as mean ± s. d, n = 6 per group; $*p < 0.05$, two-tailed Student's *t*-test.

Table S1. Primer sequences for qRT-PCR, Related to the Experimental Procedures.

Table S2. Retention times and M/Z of bile acids in UPLC-TQD-MS measurements, Related to Figure 4.

Table S3. Significantly changed metabolites in the feces, cecal content, liver, and intestine of mice exposed to TCDF.

Table S4. ^{1}H NMR chemical shifts for metabolites assigned in liver, fecal and cecal content extracts.

Table S5. Cross-validation with permutation test and CV-ANOVA for PLS-DA and OPLS-DA models from NMR spectra of fecal, cecal content, liver and intestinal extracts.

Materials and Methods

NMR-based metabolomics experiment

Sample preparation

Methanol, K_2HPO_4, NaH_2PO_4 (all in analytical grade), Sodium 3-trimethylsilyl [2,2,3,3-d4] propionate (TSP-d4) and D2O (99.9% in D) were purchased from Sigma-Aldrich (St. Louis, MO). Phosphate buffer (0.1 M K_2HPO_4/NaH_2PO_4 and PH 7.4) was prepared with K_2HPO_4 and NaH_2PO_4 for their good solubility and low-temperature stability. Liver and intestinal tissues (~50 mg) were extracted three times with 600 μL of precooled methanol-water mixture (2/1, v/v) using the PreCellys Tissue Homogenizer (Bertin Technologies, Rockville, MD). After centrifugation at 11180 x g for 10 min at 4 °C, the combined supernatants were dried. Each of the aqueous extracts was separately reconstituted into 600 μL phosphate buffer containing 50% D_2O and 0.005% TSP-d4 (chemical shift reference). Following centrifugation, 550 μL of each extract was transferred into 5 mm NMR tube. Fecal and cecal content samples were directly extracted. Briefly, samples (~50 mg) were mixed with 600 μL precooled phosphate buffer, vortexed for 30s and subjected to three consecutive freeze-thaws followed by homogenization using the Precellys Tissue Homogenizer. After centrifugation (11,180 x g, 4 °C) for 10 min, the supernatants (550 μL) were transferred into 5 mm NMR tubes for NMR analysis.

^{1}H NMR Spectroscopy

^{1}H NMR spectra of aqueous liver and fecal extracts were recorded at 298 K on a Bruker Avance III 600 MHz spectrometer (operating at 600.08 MHz for ^{1}H) equipped with a Bruker inverse cryogenic probe (Bruker Biospin, Germany). Typical one-dimensional NMR spectrum was acquired for each of all samples employing the first increment of NOESY pulse sequence (NOESYPR1D). To suppress the water signal, a weak continuous wave irradiation was applied

to the water peak during recycle delay (2 s) and mixing time (100 ms). The 90° pulse length was adjusted to approximately 10 μs for each sample and 64 transients were collected into 32 k data points for each spectrum with spectral width of 20 ppm. To facilitate NMR signal assignments, a range of 2D NMR spectra were acquired and processed for selected samples including ^{1}H-^{1}H correlation spectroscopy (COSY), ^{1}H-^{1}H total correlation spectroscopy (TOCSY), ^{1}H-^{13}C heteronuclear single quantum correlation (HSQC), and ^{1}H-^{13}C heteronuclear multiple bond correlation spectra (HMBC).

Spectral data processing and multivariate data analysis

All free induction decays (FID) were multiplied by an exponential function with a 1 Hz line broadening factor prior to Fourier transformation. ^{1}H NMR spectra were corrected manually for phase and baseline distortions and spectral region δ 0.5-9.5 was integrated into regions with equal width of 0.004 ppm (2.4 Hz) using AMIX software package (V3.8, Bruker-Biospin, Germany). Region δ 4.45-5.20 was discarded by imperfect water saturation. Regions δ 1.15-1.23 and δ 3.62-3.69 were also removed for ethanol contaminations in the cecal contents during mice dissection process. Each bucketed region was then normalized to the total sum of the spectral integrals to compensate for the overall concentration differences prior to statistical data analysis.

Multivariate data analysis was carried out with SIMCAP+ software (version 13.0, Umetrics, Sweden). Principal Component Analysis (PCA) was initially carried out on the NMR data to generate an overview and to assess data quality. Orthogonal Projection to Latent Structures with Discriminant Analysis (OPLS-DA) was subsequently conducted on the NMR data. The OPLS-DA models were validated using a 7-fold cross validation method and the quality of the model was described by the parameters R^2X and Q^2 values (Figure 5 and Supplemental Material Table S3). To facilitate interpretation of the results, back-transformation

of the loadings generated from the OPLS-DA was performed prior to generating the loadings plots, which were color-coded with the Pearson linear correlation coefficients of variables (or metabolites) using an in-house developed script for MATLAB (The Mathworks Inc.; Natwick, MA). The color-coded correlation coefficient indicates the significance of the metabolite contribution to the class separation, with a "hot" color (e.g., red) being more significant than a "cold" color (e.g., blue). In this study, a cutoff value of $|r| > 0.707$ ($r > 0.707$ and $r < -0.707$) was chosen for correlation coefficient as significant based on the discrimination significance ($p \leq 0.05$).

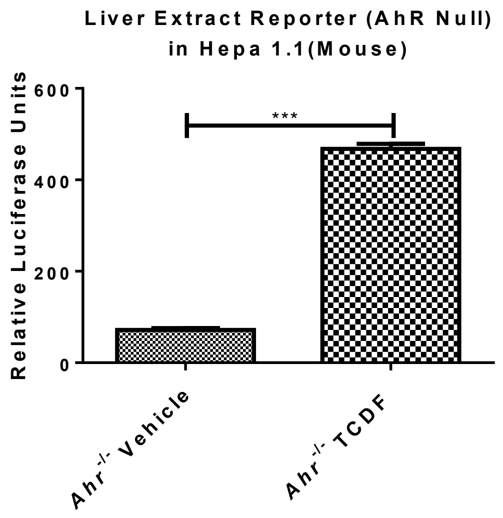

Figure S1. AHR-null liver extract reporter assay. AHR-responsiveness of extracts was examined using hepatoma reporter line, Hepa 1.1. Reporter cells were treated with 0.1 μL of control or TCDF liver extracts for 4 h. Data represent mean ± S.E.M (n=5), T-test parameters: Unpaired, Two tailed, p-value < 0.001 (***).

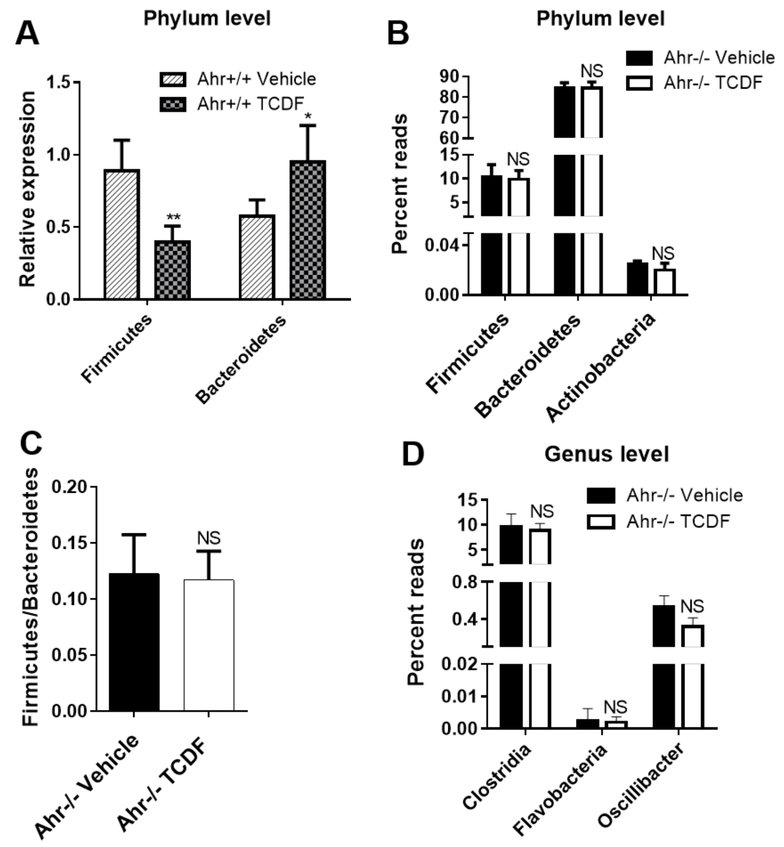

Figure S2. (A) qPCR analysis of mRNA levels of bacterial *Firmicutes* and *Bacteroidetes* in the cecal content of vehicle and TCDF-treated $Ahr^{+/+}$ mice. (B-D) 16S rRNA gene sequencing analysis at the phylum and genus level of the cecal content. Data are presented as mean ± s. d, n = 6 and 5 per group for $Ahr^{+/+}$ and $Ahr^{-/-}$ mice, respectively; $*p < 0.05$, $**p < 0.01$, NS means no significance, two-tailed Student's t-test.

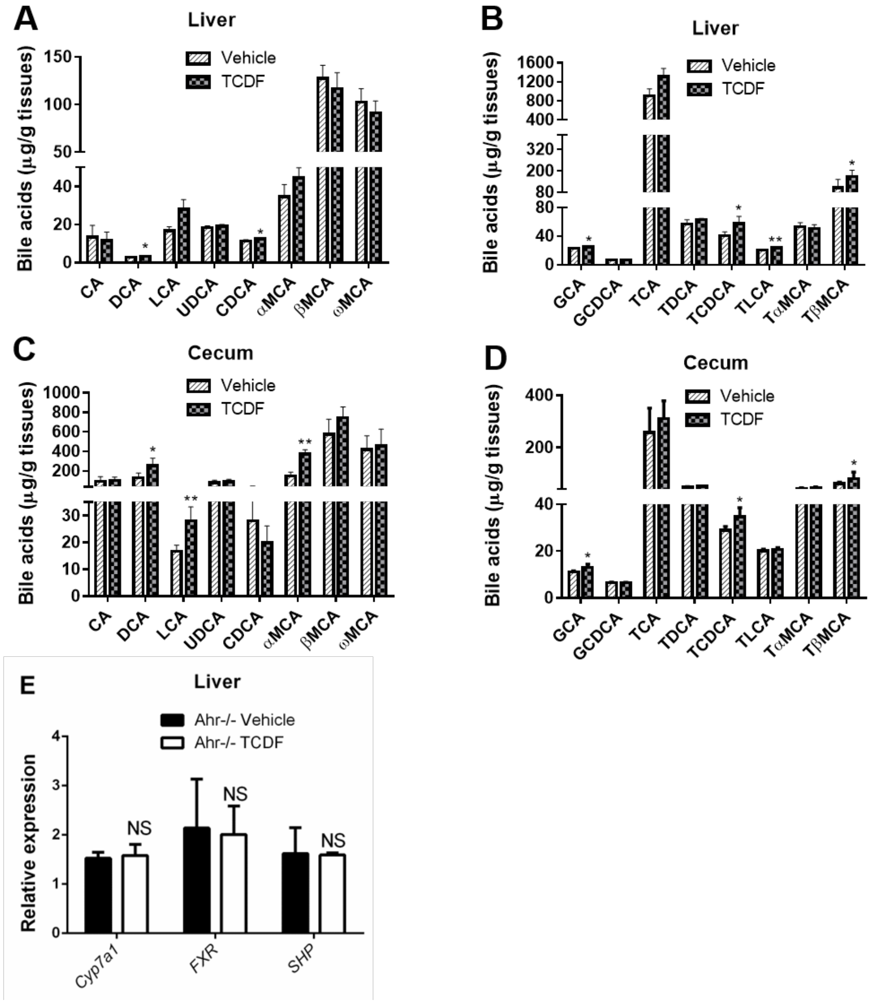

Figure S3. (A-D) Quantification of specific bile acids levels in liver and cecum of vehicle and TCDF-treated *Ahr*$^{+/+}$ mice (24 µg kg-1) by UPLC-TQMS. (E) qPCR analysis of mRNA levels of *Cyp7a1*, *Fxr* and *Shp* in the liver of vehicle and TCDF-treated *Ahr*$^{-/-}$ mice. Data are presented as mean ± s. d, n = 6 and 5 per group for *Ahr*$^{+/+}$ and *Ahr*$^{-/-}$ mice, respectively; *p < 0.05, **p < 0.01, NS, no significance, two-tailed Student's t-test. See also Table S1 and 2.

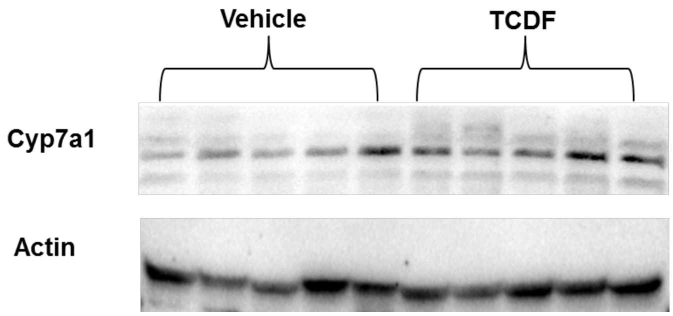

Figure S4. Western blot of Cyp7a1 and Actin levels in the liver.

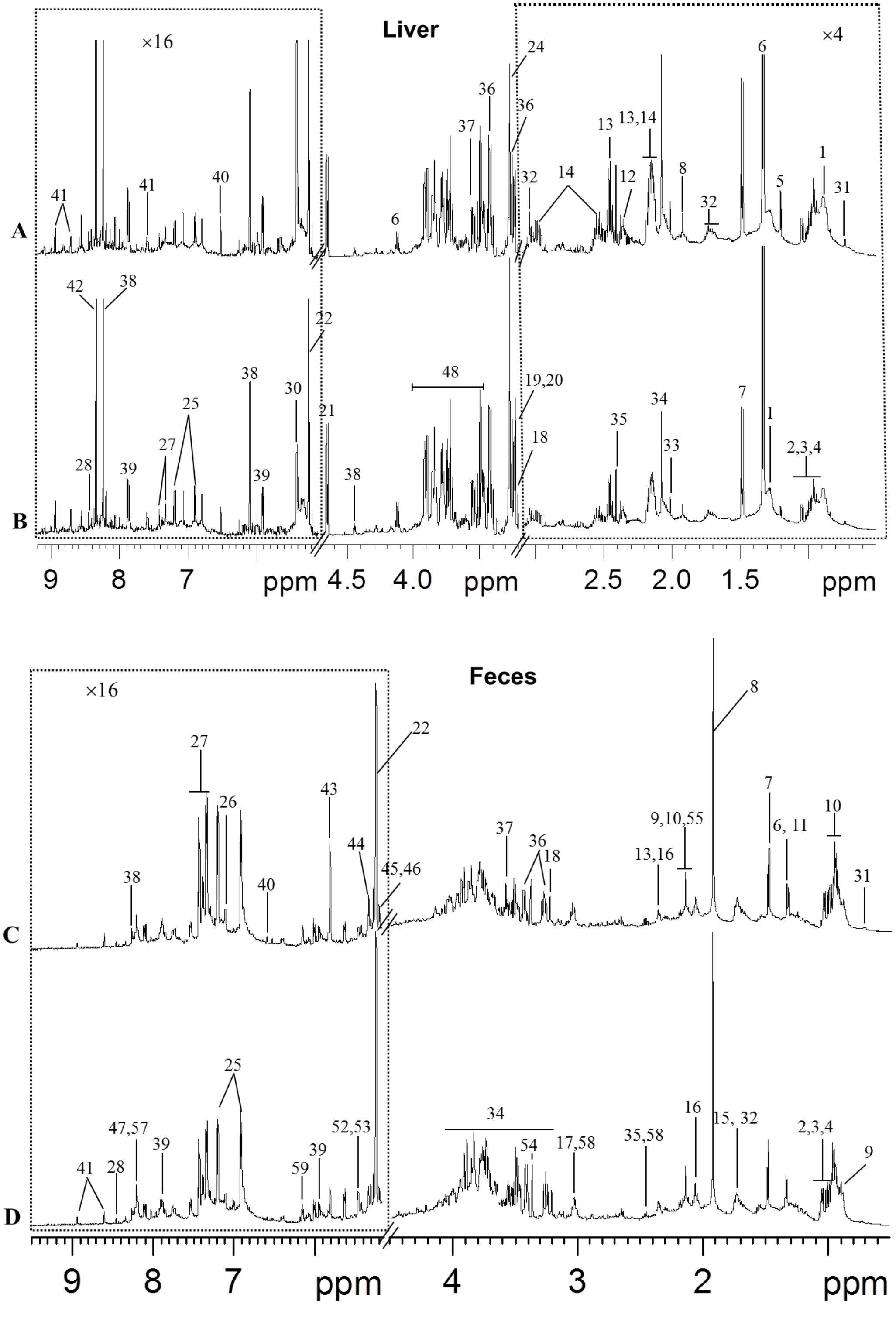
Liver
×16
×4
A
B
41
41
40
42
38
28
39
27
25
38
39
30
22
21
38
6
48
37
36
36
24
19,20
18
32
14
13
13,14
12
8
32
6
5
1
31
35
34
33
7
1
2,3,4
9
8
7
ppm
4.5
4.0
ppm
2.5
2.0
1.5
ppm
Feces
×16
C
D
27
26
38
40
43
44
45,46
22
37
36
18
13,16
9,10,55
8
7
6, 11
10
31
41
28
47,57
39
25
59
39
52,53
34
54
17,58
35,58
16
15, 32
2,3,4
9
9
8
7
ppm
4
3
2
ppm

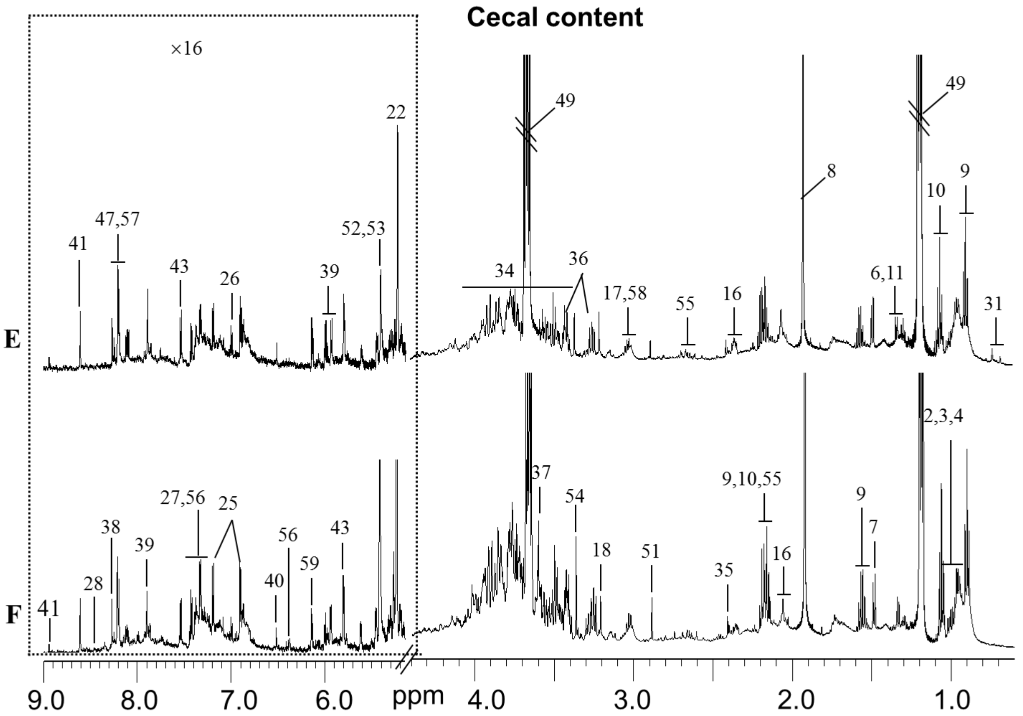

Figure S5. Representative 600 MHz ^{1}H NMR spectra of liver (A and B), fecal (C and D) and cecal content (E and F) aqueous extracts from vehicle (B, D and F) and TCDF treated group (A, C and E). The regions of δ 6.1-9.20 and δ 0.6-3.1 in the liver spectra was vertically expanded 16 times and 4 times compared with the region of δ 3.1-4.7, respectively. The regions of δ 6.1-9.4 in the fecal aqueous extracts spectra were vertically expanded 16 times compared with the region of δ 0.5-4.5. The regions of δ 6.1-9.0 in the cecal content aqueous extracts spectra were vertically expanded 16 times compared with the region of δ 0.6-4.4. Keys: 1, lipid; 2, isoleucine; 3, leucine; 4, valine; 5, D-3-hydroxybutyrate; 6, lactate; 7, alanine; 8, acetate; 9, n-butyrate; 10, propionate; 11, threonine; 12, glutamate; 13, glutamine; 14, glutathione; 15, arginine; 16, proline; 17, creatine; 18, choline; 19, phosphorylcholine; 20, glycerophosphocholine; 21, β-glucose; 22, α-glucose; 23, unsaturated fatty acid; 24, TMAO; 25, tyrosine; 26, histidine; 27, phenylalanine; 28, formate; 29, betaine; 30, glycogen; 31, bile acid; 32, lysine; 33, N-acetyl aspartate; 34, oligosaccharides; 35, succinate; 36, taurine; 37, glycine; 38, inosine; 39, uridine; 40, fumarate; 41, nicotinurate; 42, adenosine; 43, uracil; 44, α-galactose; 45, α-arabinose; 46, α-xylose; 47, hypoxanthine; 48, glucose & amino acids; 49, ethanol; 50, pyruvate; 51,TMA; 52, raffinose; 53, stachyose; 54, methanol; 56, urocanate; 57, adenine; 58, α-ketoglutarate. See also Table S4.

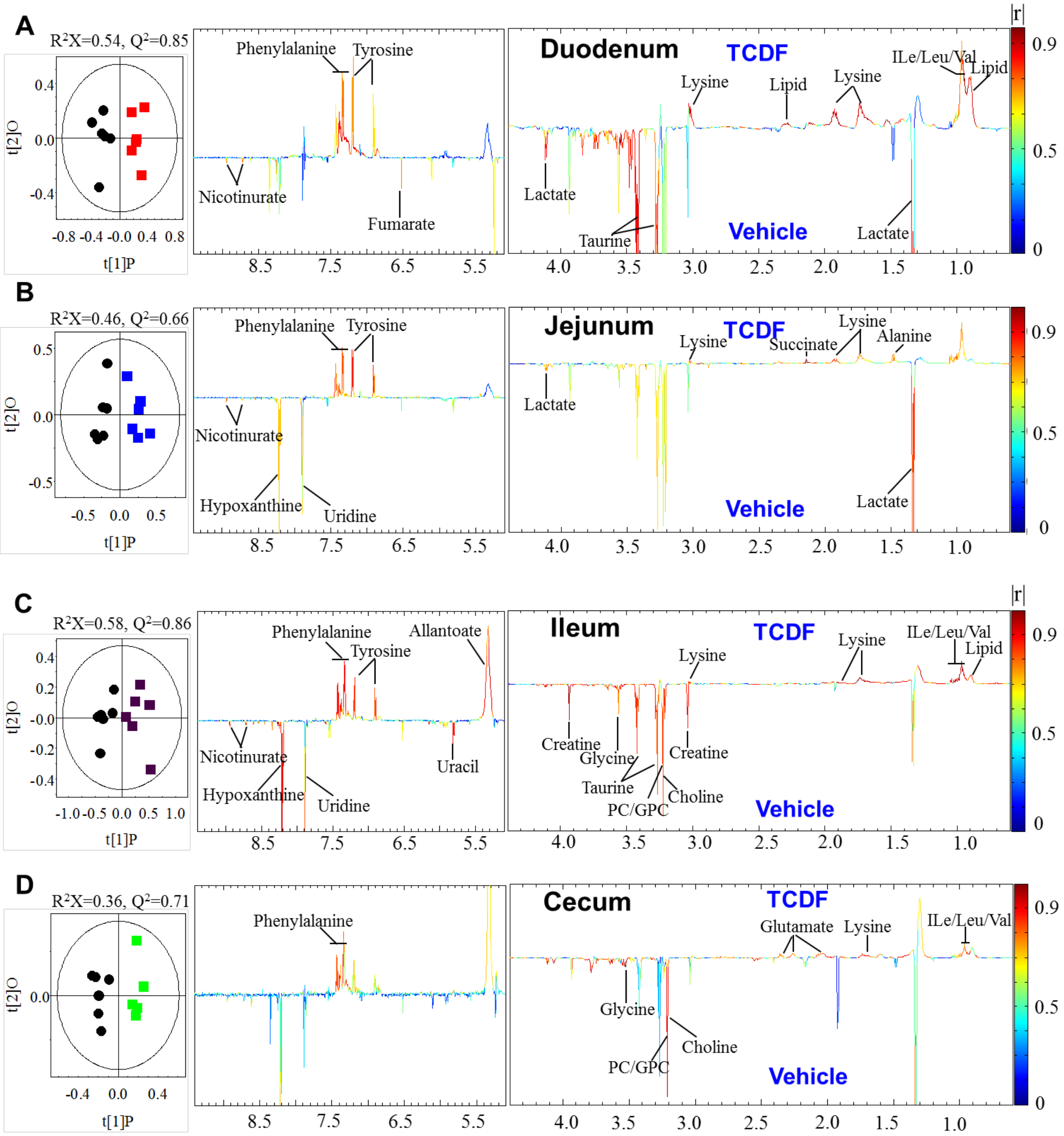
A
R²X=0.54, Q²=0.85
Phenylalanine
Tyrosine
Nicotinurate
Fumarate
Duodenum
TCDF
Vehicle
Lysine
Lipid
ILe/Leu/Val
Lactate
Taurine
B
R²X=0.46, Q²=0.66
Hypoxanthine
Uridine
Jejunum
Succinate
Alanine
C
R²X=0.58, Q²=0.86
Allantoate
Uracil
Ileum
Creatine
Glycine
Choline
PC/GPC
D
R²X=0.36, Q²=0.71
Cecum
Glutamate
t[1]P
t[2]O
|r|

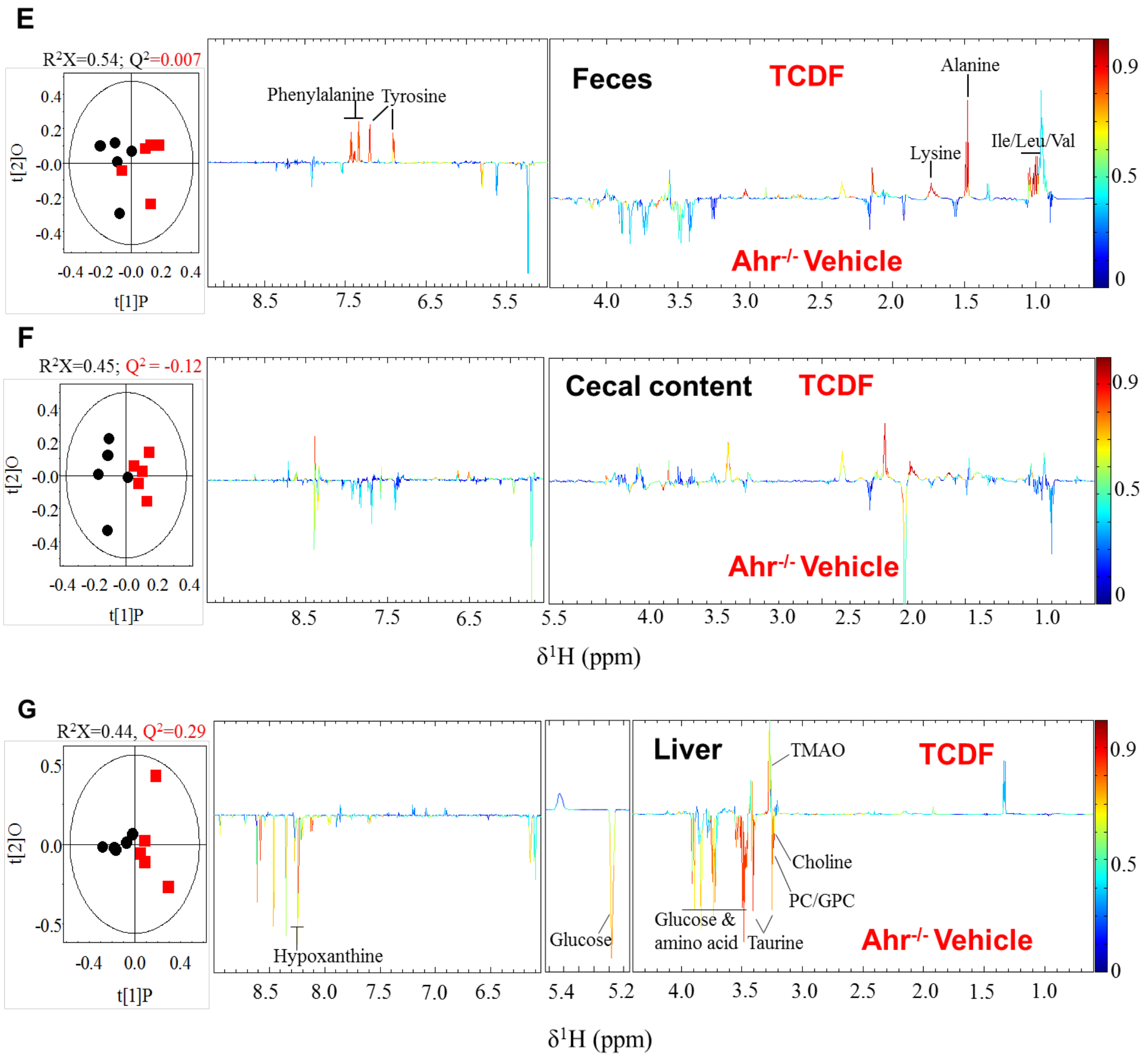

Figure S6. O-PLS-DA scores (left) and coefficient-coded loadings plots for the models (right) from NMR spectra of aqueous duodenum (A), jejunum (B), ileum (C), and cecum (D) extracts from the vehicle and TCDF-treated *Ahr*$^{+/+}$ mice and fecal (E), cecal content (F) and liver (G) extracts from vehicle and TCDF-treated *Ahr*$^{-/-}$ mice.

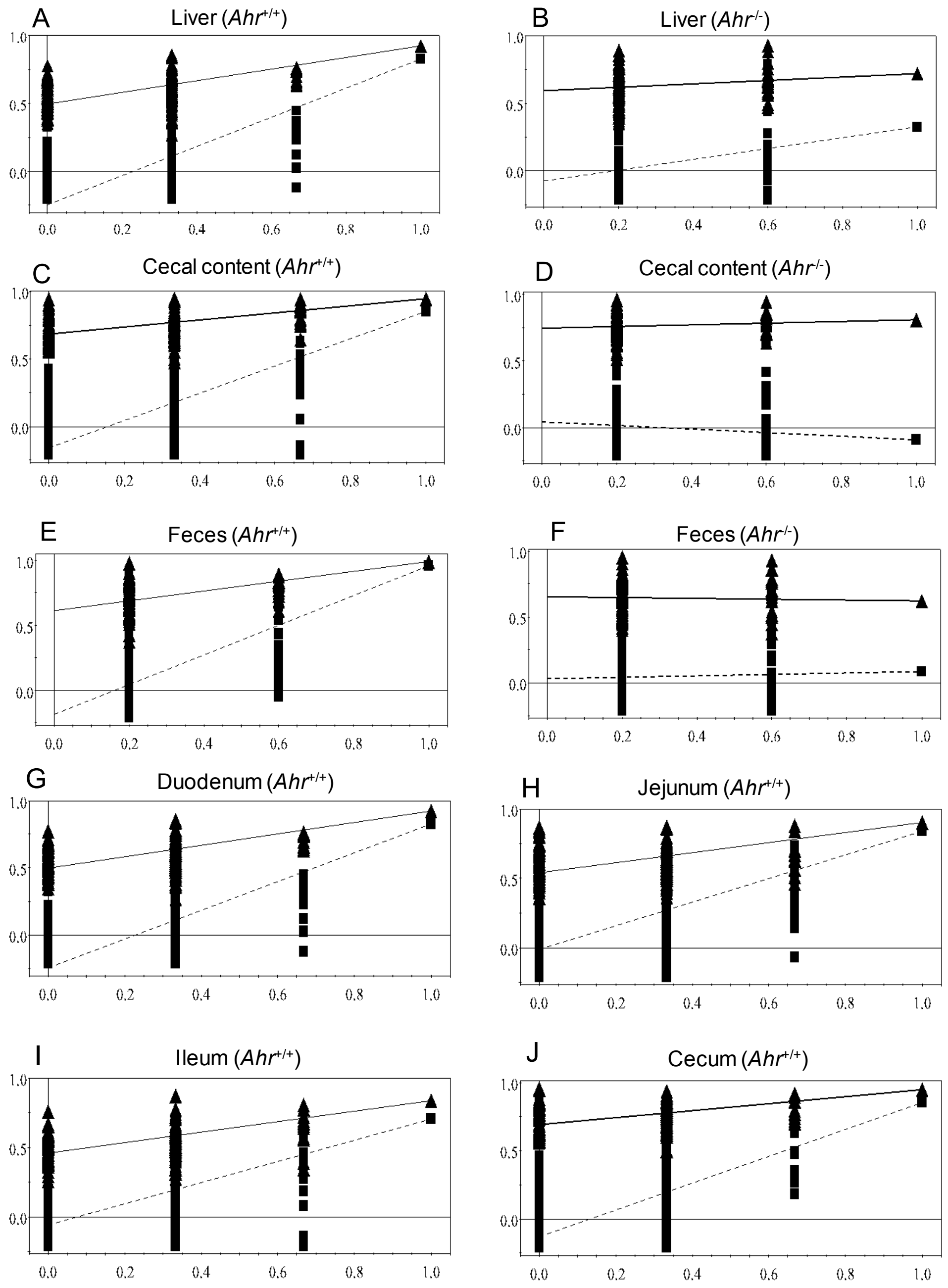

Figure S7. Cross-validation with permutations test plots (200 permutations) for the PLS-DA models constructed from ^{1}H NMR data of liver (A, *Ahr*$^{+/+}$; B, *Ahr*$^{-/-}$), cecal content (C, *Ahr*$^{+/+}$; D, *Ahr*$^{-/-}$), fecal (E, *Ahr*$^{+/+}$; F, *Ahr*$^{-/-}$), duodenum (G), jejunum (H), ileum (I), and cecum (J) aqueous extracts from vehicle and TCDF-treated mice.

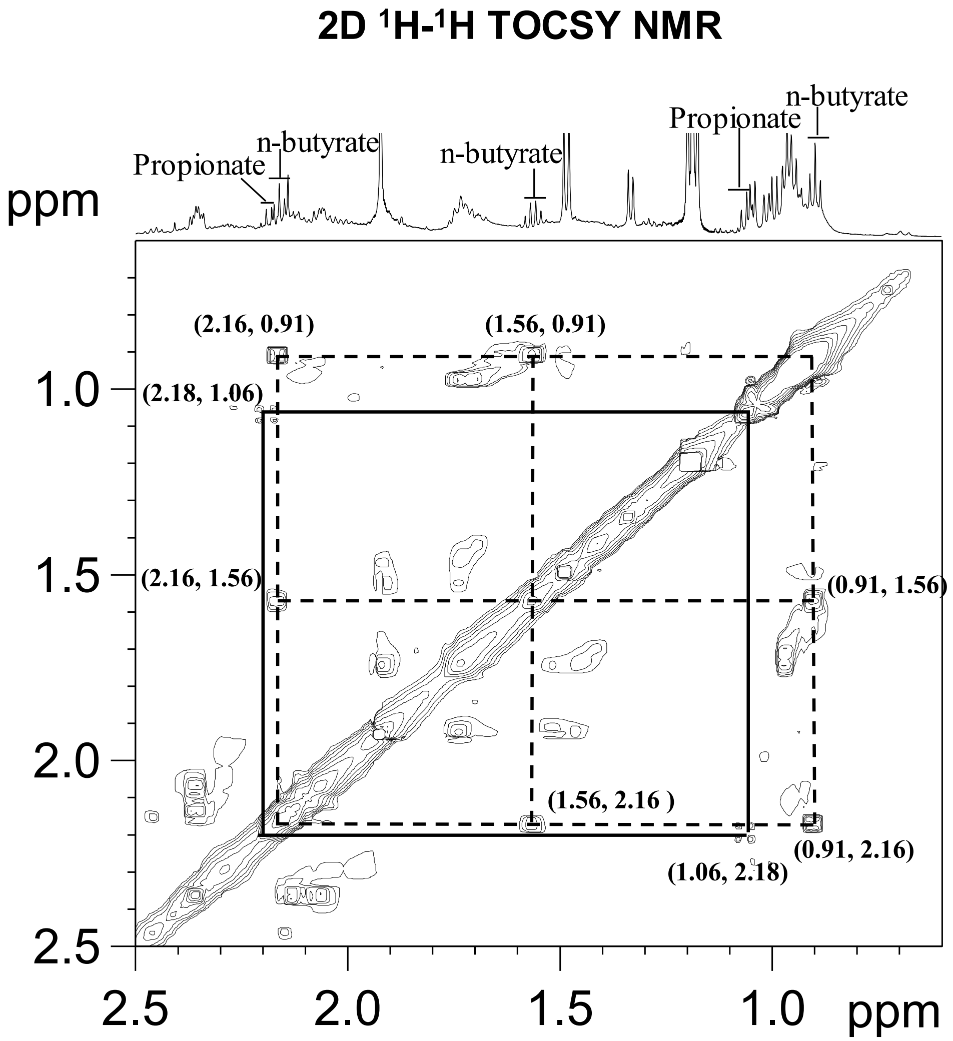

Figure S8. Two dimensional (2D) ^1H-^1H total correlation spectroscopy (TOCSY) for the identification of n-butyrate and propionate related to Figure 5A and B. The cross peaks of n-butyrate and propionate are highlighted with dotted and solid lines, respectively.

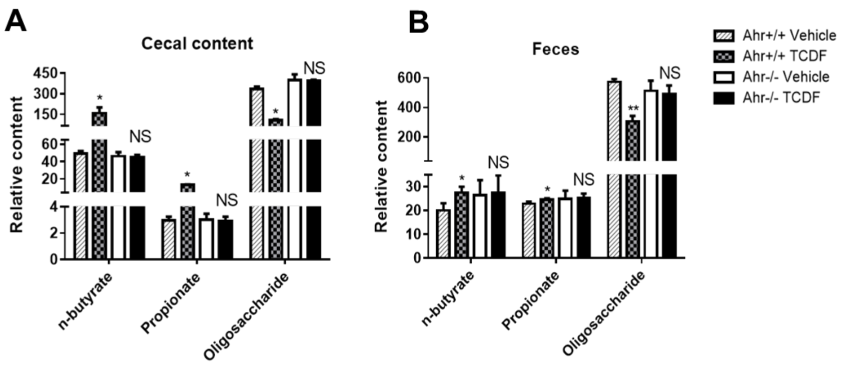

Figure S9. Measurements of n-butyrate and propionate concentration from NMR peaks integration relative to internal standard TSP in the cecal content (A) and fecal extracts (B) obtained from *Ahr*$^{+/+}$ and *Ahr*$^{-/-}$ vehicle and TCDF-treated mice. Data are presented as mean ± s. d, n = 6 and 5 per group for *Ahr*$^{+/+}$ and *Ahr*$^{-/-}$ mice, respectively; ; *p < 0.05, **p < 0.01, NS, no significance, two-tailed Student's t-test.

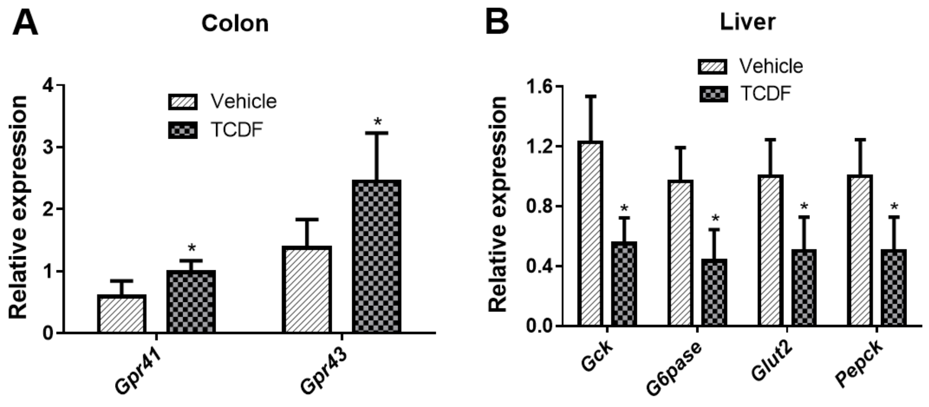

Figure S10. qPCR analysis of mRNA levels of *Gpr41* and *Gpr43* expression in the colon (A) and *Gck*, *G6pase*, *Glut2* and *Pepck* expression in the liver of $Ahr^{+/+}$ vehicle and TCDF-treated $Ahr^{+/+}$ mice. Data are presented as mean ± s. d, n = 6 per group; *$p < 0.05$, two-tailed Student's *t*-test.

Table S1. Primer sequences for qRT-PCR, Related to the Experimental Procedures.

Gene	Abbreviation	Sequence
Cytochrome P450, family 1, subfamily A, polypeptide 1	*Cyp1a1*	AGAATACGGTGACAGCCAGG<br>TTTGGGAGGAAGTGGAAGG
Cytochrome P450, family 1, subfamily A, polypeptide 2	*Cyp1a2*	AAAGGGGTCTTTCCACTGCT<br>AGGGACACCTCACTGAATGG
Cytochrome P450, family 2, subfamily E, polypeptide 1	*Cyp2e1*	CTTAGGGAAAACCTCCGCAC<br>GGGACATTCCTGTGTTCCAG
Cytochrome P450, family 2, subfamily A, polypeptide 1	*Cyp2a1*	CCCAGCAAAGAAGAGGTTCA<br>CCTTTCTCATCCACATGCAA
Aldo-keto reductase family 1 member D1	*Akr1d1*	TGCACACCACCAAATATCCCT<br>CTTCACTGCCACATAGGTCTTC
Bile acid-CoA synthetase	*Bacs*	ACCCTGGATCAGCTCCTGGAT<br>GTTCTCAGCTAGCAGCTTGG
Bile acid-CoA: amino acid *N*-acyltransferase	*Bat*	GGAAACCTGTTAGTTCTCAGGC<br>GTGGACCCCCATATAGTCTCC
Bile salt export pump (Abcb11)	*Bsep*	CTGCCAAGGATGCTAATGCA<br>CGATGGCTACCCTTTGCTTCT
Cysteine dioxygenase	*Cdo*	GGGGACGAAGTCAACGTGG<br>ACCCCAGCACAGAATCATCAG
Cysteine sulfinate decarboxylase	*Csd*	CCAGGACGTGTTTGGGATTGT<br>ACCAGTCTTGACACTGTAGTGA
Cytochrome P450, family 7, subfamily A, polypeptide 1 (Cholesterol 7α-hydroxylase)	*Cyp7a1*	AGCAACTAAACAACCTGCCAGT<br>ACTAGTCCGGATATTCAAGGATGCA
Cytochrome P450, family 7, subfamily B, polypeptide 1 (Oxysterol 7α-hydroxylase)	*Cyp7b1*	TAGCCCTCTTTCCTCCACTCATA<br>GAACCGATCGAACCTAAATTCCT
Cytochrome P450, family 8, subfamily B, polypeptide 1 (Sterol 12α-hydroxylase)	*Cyp8b1*	GGCTGGCTTCCTGAGCTTATT<br>ACTTCCTGAACAGCTCATCGG
Cytochrome P450, family 27, subfamily A, polypeptide 1 (Sterol 27-hydroxylase)	*Cyp27a1*	GCCTCACCTATGGGATCTTCA<br>TCAAAGCCTGACGCAGATG
Fibroblast growth factor 15	*Fgf15*	ACGTCCTTGATGGCAATCG<br>GAGGACCAAAACGAACGAAAT T
Short heterodimer partner	*Shp*	CGATCCTCTTCAACCCAGATG<br>AGGGCTCCAAGACTTCACACA
Farnesoid X receptor	*Fxr*	TCCAGGGTTTCAGACACTGG<br>GCCGAACGAAGAAACATGG
Myosin Vb	*Myosin Vb*	CCCCTTCTTTGTAGTCCTTGG<br>CGTACAGCGAGCTCTACACC
Protein Tyrosine Phosphatase, Receptor Type, H	*PTPRH*	GGTAAAAGTGGGTAGGAAATGGC<br>GTGGCTGTGTAGGACTGAGC
Lipocalin-2	*Lcn-2*	ATTTCCCAGAGTGAACTGGC<br>AATGTCACCTCCATCCTGGT
Segmented filamentous bacteria	*SFB*	GACGCTGAGGCATGAGAGCAT<br>GACGGCACGGATTGTTATTCA
Hepatic nuclear factor 4α1	*Hnf4a1*	AAATGTGCAGGTGTTGACCA<br>CACGCTCCTCCTGAAGAATC
Ileal bile acid-binding protein	*Ibabp*	CAGGAGACGTGATTGAAAGGG<br>GCCCCCAGAGTAAGACTGGG
Ileal bile acid transporter	*Ibat*	ACCACTTGCTCCACACTGCTT<br>CGTTCCTGAGTCAACCCACAT

Gene	Abbreviation	Sequence
Multidrug resistance-associated protein (Abcc2)	*Mrp2*	GGATGGTGACTGTGGGCTGAT<br>GGCTGTTCTCCCTTCTCATGG
Multidrug resistance-associated protein (Abcc3)	*Mrp3*	TCCCACTTTTCGGAGACAGTAAC<br>ACTGAGGACCTTGAAGTCTTGGA
Na+/taurocholate cotransporter	*Ntcp*	ATGACCACCTGCTCCAGCTT<br>GCCTTTGTAGGGCACCTTGT
Organic anion transporting protein 1	*Oatp1*	CAGTCTTACGAGTGTGCTCCAGAT<br>ATGAGGAATACTGCCTCTGAAGTG
Organic solute transporter α	*Osta*	TGTTCCAGGTGCTTGTCATCC<br>CCACTGTTAGCCAAGATGGAGAA
Organic solute transporter β	*Ostb*	GATGCGGCTCCTTGGAATTA<br>GGAGGAACATGCTTGTCATGAC
Taurine transporter	*Taut*	GCACACGGCCTGAAGATGA<br>ATTTTTGTAGCAGAGGTACGGG
Phosphoenolpyruvate carboxykinase	*Pepck*	GGCCACAGCTGCTGCAG<br>GGTCGCATGGCAAAGGG
Glucokinase	*Gck*	TAT GAA GAC CGC CAA TGT GA<br>TTT CCG CCA ATG ATC TTT TC
Glucose-6-phosphatase	*G6pase*	CTGTGAGACCGGACCAGGA<br>GACCATAACATAGTATACACCTGCTGC
Glucose transporter 2	*Glut2*	GTCCAGAAAGCCCCAGATACC<br>GTGACATCCTCAGTTCCTCTTAG
Interleukin-1 beta	*IL-1β*	GGTCAAAGGTTTGGAAGCAG<br>TGTGAAATGCCACCTTTTGA
Tumor necrosis factor alpha	*TNF-α*	AGGCTGCCCCGACTACGT<br>GACTTTCTCCTGGTATGAGATAGCAAA
Interleukin-10	*IL-10*	GGTTGCCAAGCCTTATCGGA<br>ACCTGCTCCACTGCCTTGCT
Serum amyloid A 1	*Saa1*	TCATGTCAGTGTAGGCTCGC<br>GTCTTCTGCTCCCTGCTCC
Serum amyloid A 3	*Saa3*	AGTAGGCTCGCCACATGTCT<br>TCCATTGCCATCATTCTTTG
G protein-coupled receptors	*Gpr41*	TTCTTGCAGCCACACTGCTC<br>GCCCACCACATGGGACATAT
G protein-coupled receptors	*Gpr43*	TGGTTGGACCGTGAAGACATG<br>TGGAACCTGTAATCCCAGCAC

Table S2. Retention times and M/Z of bile acids in UPLC-TQD-MS measurements, Related to Figure 4.

Bile acid	Retention time (min)	Multiple reaction mode
CA	7.98	407.2→343.2
DCA	8.95	391.3→391.3
CDCA	8.83	391.3→391.3
UDCA	8.06	391.3→391.3
αMCA	7.46	407.2→387.2
βMCA	7.57	407.2→371.2
ωMCA	7.38	407.2→387.2
GCA	7.38	464.3→464.3
GCDCA	8.03	448.3→448.3
TCA	6.68	514.3→124.0
LCA	9.98	375.3→375.3
TCDCA	7.11	498.5→124.3
TLCA	7.69	482.2→124.0
TDCA	6.63	498.5→124.3
TαMCA	6.22	514.2→107.0
T βMCA	6.28	514.2→107.0

Table S3. Significantly changed metabolites in the feces, cecal content, liver, and intestine of mice exposed to TCDF.

Metabolite	Feces R^2X=0.64 Q^2=0.88	Cecal content R^2X=0.48 Q2=0.64	Liver R^2X=0.74 Q^2=0.75	Duodenum R^2X=0.59 Q^2=0.85	Jejunum R^2X=0.48 Q^2=0.65	Ileum R^2X=0.66 Q^2=0.87	Cecum R^2X=0.56 Q^2=0.73
Lipid	—	—	+0.78[a]	+0.84	—	+0.78	—
UFA	—	—	+0.81	—	—	—	—
PUFA	—	—	+0.74	—	—	—	—
Alanine	+0.82	—	-0.83	—	+0.72	—	—
Isoleucine	+0.93	—	—	+0.81	+0.69	+0.86	+0.71
Leucine	+0.88	—	—	+0.78	+0.64	+0.83	+0.68
Valine	+0.79	—	—	+0.76	+0.64	+0.85	+0.67
Tyrosine	+0.94	+0.74	-0.73	+0.74	+0.77	+0.81	—
Phenylalanine	+0.92	+0.78	-0.72	+0.83	+0.79	+0.82	+0.84
Lysine	+0.85	—	—	+0.84	+0.71	+0.79	—
Glutamine	+0.71	—	—	—	—	—	+0.73
Glycine	—	—	-0.63	—	—	-0.72	-0.86
Glucose	-0.76	-0.79	-0.75	—	—	—	-0.63
Glycogen	—	—	+0.77	—	—	—	—
Lactate	—	—	-0.75	-0.77	-0.81	—	—
Succinate	+0.79	—	—	—	+0.84	—	—
Fumarate	—	—	—	-0.68	—	—	—
Creatine	—	—	—	—	—	-0.82	—
n-butyrate	+0.82	+0.92	—	—	—	—	—
Propionate	+0.68	+0.88	—	—	—	—	—
Taurine	—	—	—	-0.85	—	-0.84	—
Choline	-0.75	—	-0.80	—	—	-0.81	-0.82
PC/GPC	—	-0.76	-0.68	—	—	-0.85	-0.83
Inosine	—	—	-0.84	—	—	—	—
Hypoxanthine	—	—	-0.67	—	-0.65	-0.85	—
Uracil	—	—	—	—	—	-0.73	—
Uridine	—	—	—	—	-0.63	-0.66	—
Nicotinurate	—	—	-0.69	—	-0.67	-0.68	—
Allantoate	—	—	—	—	—	+0.78	—
Oligosaccharides	-0.81	-0.71	—	—	—	—	—

[a]Correlation coefficient values obtained from OPLS-DA of treatment groups.

+ and – indicate a significant increase and decrease of metabolite levels in the treatment groups compared to the control mice; — no change.

Table S4. ^{1}H NMR chemical shifts for metabolites assigned in liver, fecal and cecal content extracts.

Key	Metabolites	Moieties	δ ^{1}H (ppm) and multiplicity[a]	Samples[b]
1	Lipid	CH_3, $(CH_2)_n$, CH_2-C=C, CH_2-C=O,C-CH_2-C=,-CH=CH-	0.89(m), 1.27(m), 2.0(m), 2.3(m), 2.78(m), 5.3(m)	L
2	Isoleucine	αCH, βCH, γCH_3, δCH_3	3.65(d), 1.95(m), 0.99(t), 1.02(d)	L, F, C
3	Leucine	αCH, βCH_2, γCH_3, δCH_3	0.94(d), 3.72(t), 1.96(m), 0.91(d)	L, F, C
4	Valine	αCH, βCH, γCH_3	3.6(d), 2.26(m), 0.98(d), 1.04(d)	L, F, C
5	D-3-hydroxybutyrate	CH, CH_2, γCH_3, CH_2	4.16(dt),2.41(dd),1.20(d),2.31(dd)	L
6	Lactate	αCH, βCH_3	4.11(q), 1.32(d)	L, F, C
7	Alanine	αCH, βCH_3	3.77(q), 1.48(d)	L, F, C
8	Acetate	CH_3	1.91(s)	L, F, C
9	n-butyrate	CH_3, CH_2, CH_2	0.91(t), 1.56(m), 2.16(t)	F, C
10	Propionate	CH_3, CH_2	1.06(t), 2.18(q)	F, C
11	Threonine	γCH_3, αCH, βCH	1.33(d), 3.59(d), 4.26(m)	F, C
12	Glutamate	αCH, βCH_2, γCH_2	2.08(m), 2.34(m), 3.75(m)	L
13	Glutamine	αCH, βCH_2, γCH_2	2.15(m), 2.44(m), 3.77(m)	L, F
14	Glutathione	CH_2, CH_2, S-CH_2, N-CH, CH	2.16(m), 2.55(m), 2.95(dd), 3.78(m), 4.56(q)	L
15	L-arginine	γCH_2, βCH_2, αCH	1.72(m), 1.93(m), 3.77(m)	F
16	L-proline	CH_2, CH_2, CH	2.05(m), 2.34(m), 3.4(m)	F, C
17	Creatine	CH_3, CH_2	3.03(s), 3.93(s)	F, C
18	Choline	$N(CH_3)_3$, OCH_2, NCH_2	3.2(s), 4.05(t), 3.51(t)	L, F, C
19	Phosphocholine (PC)	$N(CH_3)_3$, OCH_2, NCH_2	3.22(s), 4.21(t), 3.61(t)	L
20	Glycerophosphocholine	$N(CH_3)_3$, OCH_2, NCH_2	3.22(s), 4.32(t), 3.68(t)	L
21	β-Glucose	1-CH	4.66(d)	L
22	α-Glucose	1-CH	5.23(d)	L, F, C
23	Unsaturated fatty acid	CH=CH	2.73, 5.3	L
24	TMAO	CH_3	3.27(s)	L
25	Tyrosine	CH, CH	6.89(dd), 7.18(dd)	L, F, C
26	Histidine	2-CH, 4-CH, CH_2	7.75(t), 7.08(d), 6.05(d)	L, F, C
27	Phenylalanine	Ring-CH	7.40(m), 7.33(m), 7.35(m)	L, F, C
28	Formate	CH	8.45(s)	L, F, C
29	Betaine	CH_2, CH_3	3.27(s), 3.93(s)	L
30	Glycogen	1-CH	5.38-5.45(m)	L
31	Bile acid	CH_3	0.73(m)	L, F, C
32	Lysine	αCH, βCH_2, γCH_2, δCH_2	3.76(t), 1.89(m), 1.72(m), 3.01(t)	L, F, C
33	N-acetyl aspartate	CH_3	2.01(s)	L
34	Oligosaccharides	αCH resonances	3.3-3.9	F, C
35	Succinate	CH_3	2.41(s)	L, F, C
36	Taurine	S-CH_2, N-CH_2	3.26(t), 3.40(t)	L, F, C
37	Glycine	CH_2	3.57(s)	L, F, C
38	Inosine	14-CH, 1-CH, 8-CH, 4'-CH, 5'-CH, CH_2(1/2), CH_2(1/2)	8.34(s), 6.09(d), 8.22(s), 4.76(t), 4.47(m)	L
39	Uridine	11-CH, 7-CH, 12-CH, 6-CH, 5-CH, 4-CH, CH_2, CH_2	7.88(d), 5.92(d), 5.9(d), 4.36(m), 4.24(t)	L, F, C
40	Fumarate	CH	6.53(s)	L, F, C
41	Nicotinurate	2-CH, 6-CH, 4-CH, 5-CH	8.93(s),8.62(d), 8.25(d),7.60(dd),	L, F, C

Key	Metabolites	Moieties	δ ^{1}H (ppm) and multiplicity[a]	Samples[b]
42	Adenosine	14-CH	8.32(s)	L, C
43	Uracil	1-CH, 2-CH	5.81(d), 7.54(d)	L, F, C
44	α-galactose	1-CH, 2-CH, 3-CH	5.28(d), 3.81(dd); 3.97(m)	F
45	α-arabinose	1-CH, 2-CH	5.21(d), 3.87(dd)	F
46	α-xylose	1-CH, 2-CH, 3-CH	5.20(d), 3.53(dd), 3.68(m)	F
47	Hypoxanthine	1-CH, 2-CH	8.20(s), 8.21(s)	F, C
48	Glucose & amino acids	αCH resonances	3.3-3.9	L
49	Ethanol	CH_3, CH_2	1.18(t), 3.65(q)	C
50	Pyruvate	CH_3	2.38(s)	F, C
51	TMA	CH_3	2.88(s)	F, C
52	Raffinose	1-CH	5.41(d)	F, C
53	Stachyose	1-CH	5.41(d)	F, C
54	Methanol	CH_3	3.36 (s)	F, C
55	Methionine	δCH_3, βCH_2, γCH_2	2.14(s), 2.16(m), 2.65(t)	F, C
56	Urocanate	CHCOOH, CH(ring), 5CH	6.40(d), 7.31(d), 7.43(s)	F, C
57	Adenine	2CH, 6CH	8.19(s), 8.21(s)	F, C
58	α-ketoglutarate	γCH_2, βCH_2	2.45(t), 3.01(t)	F, C

[a]Key: s, singlet; d, doublet; t, triplet; q, quartet; m, multiplet; dd, doublet of doublet. [b]Liver (L), fecal (F) and cecal content (C) aqueous extracts.

Table S5. Cross-validation with permutation test and CV-ANOVA for PLS-DA and OPLS-DA models from NMR spectra of fecal, cecal content, liver and intestinal extract.s

	Ahr$^{+/+}$ **TCDF Vs *Ahr***$^{+/+}$ **Vehicle**		***Ahr***$^{-/-}$ **TCDF Vs *Ahr***$^{-/-}$ **Vehicle**	
**Samples**	**OLPS-DA CV-ANOVA**	**PLS-DA Permutation test**	**OLPS-DA CV-ANOVA**	**PLS-DA Permutation test**
Feces	***	√	NS	×
Cecal content	*	√	NS	×
Liver	**	√	NS	×
Duodenum	***	√	—	—
Jejunum	*	√	—	—
Ileum	***	√	—	—
Cecum	**	√	—	—

*p < 0.05, **p < 0.01, ***p < 0.001, NS, no significance, √ pass, × fail, — not determined.
